# Comparative morpho-anatomical standardization and chemical profiling of root drugs for distinction of fourteen species of family Apocynaceae

**DOI:** 10.1186/s40529-022-00342-z

**Published:** 2022-04-25

**Authors:** Pankaj Kumar, Anil Bhushan, Prasoon Gupta, Sumeet Gairola

**Affiliations:** 1 Plant Sciences & Agrotechnology Division, CSIR-Indian Institute of Integrative Medicine, Canal Road, Jammu, 180001 Jammu and Kashmir India; 2 Natural Products and Medicinal Chemistry Division, CSIR-Indian Institute of Integrative Medicine, Canal Road, Jammu, 180001 Jammu and Kashmir India; 3 Academy of Scientific and Innovative Research (AcSIR), Ghaziabad, 201002 Uttar Pradesh India

**Keywords:** Apocynaceae, Root drug samples, Adulteration, Chemical profilling, LC–MS profiling, Reference standards

## Abstract

**Background:**

The root drugs of the family Apocynaceae are medicinally important and used in Indian Systems of Medicine (ISM). There is often a problem of misidentification and adulteration of genuine samples with other samples in the market trade. Keeping in view the adulteration problem of raw drug material, comparative macroscopic and microscopic (qualitative and quantitative) characterisation and chemical analysis (TLC and LC–MS profiling) of a total of 14 economically important root drugs of family Apocynaceae were done for practical and rapid identification. A total of 33 qualitative botanical characteristics of root samples were subjected to Principal Component Analysis (PCA) and Cluster analysis to identify taxonomically significant characteristics in the distinction of root drug samples at the species level.

**Results:**

Comparative qualitative and quantitative data on morphological, macroscopic, and microscopic characters were generated for the studied 14 species. Despite the similarity in some root characters, a combined study involving the surface, anatomical, and powder features helped distinguish root samples at the species level. The relative relationship between selected species was represented as clustering or grouping in the dendrogram. PCA analysis determined significant characters leading to species grouping and identification. Results showed that clustering of xylem vessels in cross-section, pore size, and distribution in the cut root, the shape of starch grains, the thickness of cork zone were among the most notable characters in species distinction. Chemical profiling revealed unique fingerprints and content of chemical compounds, which were significant in identification of root drug samples.

**Conclusions:**

The comparative botanical standards and chemical profiles developed in the present study can be used as future reference standards for the quick, easy, and correct identification of root drug samples to be used in the herbal drug industry. Further, the identified significant microscopic characters have the potential for taxonomic studies in species delimitation.

**Supplementary Information:**

The online version contains supplementary material available at 10.1186/s40529-022-00342-z.

## Background

The global herbal market is very fast-growing, with large numbers of herbal products launched in the market every year. Overall international trade in medicinal plants and their products in 2010 was US$ 60 billion, which is expected to reach US$ 5 trillion by 2050 (Nirmal et al. 2013). India is known as the second-largest exporter of medicinal plants after China (Dhanabalan 2011). Around 960 medicinal plants are traded in India, of which 178 are known with high trade value with annual consumption of more than 100 metric tonnes (NMPB 2010). The Apocynaceae, belonging to the order Gentianales, also known as ‘Dogbane family’ or ‘Toxic plant’s family,’ is considered one of the largest and economically most important angiosperms family. It comprises about 5100 species belonging to 366 genera in five subfamilies, plants are generally trees, shrubs, and vines distributed mainly in tropical and subtropical regions, with several genera widely occurring in various regions of India (Endress and Bruyns 2000; Lens et al. 2009; Nazar et al. 2013; Endress et al. 2014; eFI 2020). Plants of the family Apocynaceae are characterized by latex and are rich in several metabolites, such as alkaloids, triterpenoids, flavonoids, steroids, phenols, lactones, and glycosides (Hofling et al. 2010; Bhadane et al. 2018). Plants of this family possess many pharmacological properties (Endress 1997; Yarnell and Abascal 2002; Bhadane et al. 2018). Roots of several Apocynaceae species are widely used in Indian Systems of Medicine (ISM) such as Ayurveda, Siddha, and Yunani systems (Khare 2007; Devi et al. 2017; Jeewandara et al. 2017). Only a few selected species are cultivated commercially, and most of the traded raw plant material is collected from wild sources. Due to widespread use in Indian traditional medicines and the similar appearance of plants, several species of Apocynaceae are often prone to adulteration (Devi et al. 2017).

There are identification problems with raw herbal root drugs of Apocynaceae due to similar or confusing names, similar physical appearance, lack of an organized plant collection and procurement chain. The use of the wrong species for medicinal purposes can be harmful to end-users. Correct identification and authentication of herbal drug samples are essential to ensure traditional medicines’ efficacy, purity, and quality (Sahoo et al. 2010). Botanical and chromatographic fingerprint, reference standards are helpful to identify and to determine the purity and quality of herbal drugs (Zafar et al. 2010; Folashade et al. 2012; Upton et al. 2020). For identification and ensuring consistent quality of plant raw materials and botanicals of herbal products, several chemical identification methods (qualitative and quantitative) are accepted.

Reference standards are helpful in the correct identification and distinction of different root drug samples. Microscopic methods are known to be taxonomically significant for the identification of fragmented herbal samples and are used for sample identification in the various traditional pharmacopeia, for taxonomic characterization and systematic studies in many plants (Kraemer 1920; Metcalfe and Chalk 1957; Carlsward et al. 1997; Scatena et al. 2005; Aldasoro et al. 2005; Matias et al. 2007; Figueroa et al. 2008; Zarrei et al. 2010; Ginko et al. 2016). Botanical identification by macroscopic and microscopic studies of herbal plants is known to be simple and easy (Apraj et al. 2011). In chemical based identification, Thin Layer Chromatography (TLC) remains the simplest, efficient, with low cost and rapid tool to check and identify known markers compounds in plant extract (Pascual et al. 2002). TLC is a common chromatographic technique for separating non-volatile substances and quite valuable for assessing quality of herbal remedies (Yuen and Lau-Cam 1985). Michael Tsweet was the first to introduce the separation and identification of plant constituents using chromatography (Ettre and Sakodynskii 1993). Now a day, chromatographic and spectroscopic techniques are used for the quantitative estimation and quality control of herbal drugs (Balekundri and Mannur 2020).

Considering the medicinal and trade value and the authentication problem of raw drug material, root drug samples of 14 species of family Apocynaceae were selected for the present study. The selected plants are used in different Ayurvedic formulations, reported with high estimated annual trade value, and often have adulteration problems. The present study aims to develop a detailed comparative morphological, macroscopic, and microscopic standard (qualitative and quantitative) along with chemical profiling for practical and rapid identification of the highly traded fourteen root drugs of the family Apocynaceae.

## Material and methods

### Plant material

For the present study, authentic dry raw root drug samples (RDS) of the fourteen species of the family Apocynaceae available at Crude Drug Repository (CDR) were selected. CDR is a national referral facility (a sub-section of Janaki Ammal Herbarium (RRLH) at CSIR-IIIM, Jammu, which is an internationally recognised Herbarium), having a collection of > 4200 authentic raw plant drug specimens collected from different parts of India. Accepted botanical names and synonyms of the selected species were verified from theplantlist.org (TPL 2013).

### Botanical studies

Surface characters of root drug samples (such as color, texture, appearance, nature, etc.) and transverse cut root surface characters (such as surface appearance, color, thickness, and nature of various zones) were analyzed by hand lens and by stereomicroscope (Leica S9i).

For the anatomical study, dry raw root drug samples (RDS) were kept in FAA fixative; Formalin (5 ml) + Acetic acid (5 ml) + 70% Ethyl alcohol (90 ml), for 24 h and then in water for softening and rehydrating the tissues. Three to five root specimens (each of nearly the same size) were studied for anatomical study. Thin transverse sections (T.S.) were obtained by freehand sectioning using a razor blade. Thin sections were serially dehydrated and stained, according to Berlyn and Miksche (1976), with modifications in some steps. Sections were initially dehydrated in 50% and then 70% alcohol (each for 10 min), stained in safranin stain (5–10 min), decolorized in 70% alcohol (5 min), stained in fast green (2–3 min), decolorized in 70% alcohol (5 min), dehydrated in 90% alcohol and absolute alcohol (each for 2 min) and finally cleared in xylene for 1–2 min. Xylene cleared sections were carefully mounted in Canada balsam and then observed under the compound light microscope.

In powder study, crushed dried root drug samples were characterized for organoleptic characters (color, odor, taste, and texture) and microscopic characters (cell types and cell contents). An iodine test was performed in root powder and T.S. of the root to study the shape and size of starch grains. Microscopic characters were observed using a compound microscope (LEICA DM 750) with an associated camera (LEICA ICC50E). Histological measurements were also done for various tissue zones, cells, and cell contents using Leica software (LEICA LAS V 4.9.0 software).

### Statistical analysis

The botanical data were subjected to variance analysis, Principal Component Analysis (PCA), and Cluster analysis. Variance analysis of selected quantitative characters was done using descriptive statistics such as mean and standard deviation by Tukey’s post hoc test using Minitab 17 (Minitab, LLC, State College, PA, USA). Among various studied root botanical characters, a total of 33 qualitative macroscopic and microscopic characters (Table 1) appearing in more than one state were selected, coded in binary (20 characters), and multistate (13 characters) numerical values for creating a data matrix (Additional file 1: Table S1). Selected root characters, their types, and codes for identifying the studied RDS are given in Table 1. Botanical traits were subjected to PCA and Cluster analysis with Paleontological Statistics Software (PAST) (Version 3.26) to study species grouping and determine the taxonomically significant characters for species grouping (Hammer et al. 2001). Cluster analysis was done by Ward’s hierarchical clustering method based on Euclidean metric distances. Results of PCA are presented as two-dimensional scatter plots representing species and character states.

**Table 1:** Some important root characters, their types, and codes for identification of the studied RDS of family Apocynaceae used in ISM

S.No	Characters	Types (code)
Raw drug surface (Figs. 1, 2)		
1	Surface appearance	Nearly smooth and uniform bark (1)/ Bark may be rough or scaly (2)
2	Surface wrinkles	Present (1)/ Absent (2)
3	Surface fissures	Present (1)/ Absent (2)
4	Granular or powdery mass on scrapping	Present (1)/ Absent (2)
5	Nodule like surface protuberances	Present (1)/ Absent (2)
6	Transverse surface cracks	Present (1)/ Absent (2)
7	Surface texture	Hard (1)/ Soft (2)
	Raw drug cut root (Figs. 1, 2)	
8	Cork color	Dark brown (1)/ Light brown (2)
9	Cortex colour	Dark brown (1)/ Light brown (2)/ Light cream (3)
10	Bark nature	Separated from the main root portion (1)/ Adhered to main root portion (2)
11	Pores in the woody part	Uniformly distributed (1)/ Uneven (2)
12	Pore arrangement in the woody part	Circular arrangement (1)/ Spoke like arrangement (2)/ Random (3)/Circular and spoke like both (4)
13	Pore size and distribution in the woody part	Large-sized, uniformly present (1)/ Large-sized, scattered (2)/ Small-sized, uniformly present (3)/ Small-sized, scattered (4)/ Very small (5)
14	The appearance of pore size in the woody part	Nearly uniform size (1)/ Varying pore (2)
Transverse section (Figs. 1, 2)		
15	Sclereids in bark	Present (1)/ Absent (2)
16	Secretory canals in the bark	Well-formed (1)/ Deformed (2)
17	Crystals in bark	Abundant (1)/ Rare (2)
18	Cork zone thickness (percent of the total root thickness)	Thick [> 10%] (1)/ Medium [5*–*10%] (2)/ Thin [< 5%] (3)
19	Cork lignification	Lignified (1)/ Less lignified (2)/ Parenchymatous (3)
20	Cortex zone thickness (percent of the total root thickness)	Thin [< 20%] (1)/ Medium [20*–*30%] (2)/ Thick [> 30%] (3)
21	Clustering of xylem vessels	Solitary (1)/ Groups (2)/ Linear (3)/ Groups and linear both (4)/ Solitary and groups (5)/ Solitary and linear (6)/ Solitary, Linear and in groups (7)
22	Xylem zone thickness (percent of the total root thickness)	Thin [40*–*60%] (1)/ Medium [60*–*80%] (2)/ Thick [> 80%] (3)
23	Medullary rays appearance	Distinguished (1)/ Less distinguished (2)
24	Width of medullary rays	Uniform width throughout (1)/ one-celled at the center and slightly widened at outer region (2)/ Comparatively much wider at outer region than the center region (3)/ Less distinguished (4)
25	Annular ring markings (in cut root surface)	Distinct (1)/ Indistinct or Absent (2)
26	Pith	Present (1)/ Absent (2)
27	Iodine test of dry T.S. showing an abundance of starch in	Cork (1)/ Cortex (2)/ Medullary rays (3)/ Both cortex and medullary rays (4)
Powder characters (Additional file 1: Fig. S1, S2)		
28	The shape of starch grains	Spherical (1)/ Oval (2)/ Slightly oval to elongated (3)/ More than two shapes (4)/ Oval to spherical shaped (5)
29	Grouping of starch grains	Single (1)/ Two to four units (2)/ More than four units (3)
30	Crystal type	Prismatic (1)/ Rosette (2)/ Both Prismatic & Rosette (3)/ Prismatic, Rosette & Acicular (4)
31	Colored fragments	Few (1)/ Abundant (2)
32	Sclereids	Few (1)/ Abundant (2)
33	Cork cells	Few (1)/ Abundant (2)

### Chemical identification

#### Extraction procedure for chromatographic fingerprinting

The root samples were air dried at temperature 25 °C ± 2 °C and relative humidity of 65% ± 5%. The dried material was powdered using pestle and mortar. The 10.0 gm of dried powdered material was socked in methanol, kept under sonication for 2 h, and kept overnight. A similar extraction procedure was repeated 24 h with the same solvent until a clear and colorless solvent was obtained. The combined extract was then filtered through Whatman filter paper (No.2) and dried under a vacuum evaporator at 40 °C. Dried extract was stored at 0 °C in an airtight container until used.

#### Thin Layer Chromatography (TLC) fingerprint

In present study, TLC profiles were developed for the root samples of the selected species. For the TLC fingerprint, methanolic extract of samples was used. The sample (2 gm) was dissolved in 10 ml methanol with continuous stirring at room temperature for 24 h. The extract was filtered through Whatman filter paper No. 2. Subsequently, the extract was diluted (1 ml extract) in 25 ml of methanol and was later used for TLC fingerprinting. The root extract was spotted with a capillary tube onto a silica-gel TLC plate with F_254_ fluorescent indicator and developed in suitable solvent polarity for resolution (Factor 1991). The developed plates were then stained in anisaldehyde reagent and heated at 105 °C for 5 min. The movement of the active compound was expressed and recorded by the retention factor value (R_*f*_).

For development of TLC, methanolic crude extract (10 µl) of root samples (100 mg in 10 ml) was applied on to a silica-gel TLC plate with F254 fluorescent indicator. Prior to chromatography, the chamber was saturated with mobile phase for 15 min. The loaded plate was placed in a developing chamber with a mobile phase until the mobile phase rose to 7 cm in height. The TLC was developed in varied solvent combinations for each plant (Table 6). The developed plate was air-dried to remove the solvent from the container, stained with anisaldehyde reagent, heated at 105 °C for 5 min and then examined at white light for the varied band patterns.

#### Adsorbent

Chromatographic Silica gel F_254_ mixture with an average particle size of 5 µm.

#### Application volume

10 µl each of the sample solution as 7-mm bands.

Relative humidity: Condition the plate to a relative humidity of about 33% using a suitable device.

Developing distance: 7 cm.

Derivatization reagent: Anisaldehyde reagent: add 20 ml of acetic acid and 10 ml of sulphuric acid to 170 ml of cold methanol and mix well. After cooling to room temperature, add 1 ml of anisaldehyde to the mixture.

### LC–MS analysis

The chemicals used for the LC–MS analysis were MS-grade acetonitrile, water, acetic acid, and formic acid; all were purchased from Merck, Germany. Other solvents and chemicals used for the extraction were of analytical grade and procured from Merk, Germany.

The sample for LC–MS analysis was prepared in a volumetric flask in methanol–water (1:1, v/v). The crude extract was filtered through a 0.25 μm disposal membrane filter (Millipore) and made appropriate dilutions using methanol. The stock and working solution were stored at + 4 °C. An Agilent 1260 liquid chromatography system (Agilent, USA) equipped with a quaternary solvent delivery system, an autosampler, and a column heater was used. The chromatographic separation was performed on Merck Chromolith fast gradient RP18_e_ column (100 mm × 4.6 mm) protected by a Chromolith guard column. The mobile phase consisted of A (0.1% aq. formic acid: 1.0% ACN, v/v/v) and B (Acetonitrile). A gradient elution was performed with mobile phase started with B-0%; 4.0 min B-20%; 15 min B 50%; 20 min B-50%; 25 min B 70%; 35 min B-70% 38 min B-85%; 42 min B-85%; 45 min B-0% and at 47 min B-0%. The flow rate was monitored at 0.5 mL/min. The injection volume was 1 μL, and the column temperature was maintained at 30^0^C. A 6410B triple quad LC/MS system from Agilent was used to detect a hybrid triple quadrupole mass spectrometer equipped with Turbo V sources. The analyses were performed using electrospray ionization (ESI) sources in positive and negative modes. The operation conditions were as follows: scan range of 110–1300 amu, V charging 4000 V, ion source temperature 300^0^C, nebulizer 50psi, gas flow 13L/min, capillary voltage 4000, and a step size of 0.1 amu. Nitrogen was used in all cases. Agilent Mass Hunter software (version B.04.00) was used for data acquisition and processing.

## Results

The scientific literature on taxonomic, medicinal, and commercial aspects was searched from various sources such as scientific journals, edited books, floras, scientific databases, eFloras, online databases, etc. Raw root drug samples of selected species in the present study are essential ingredients in different Ayurvedic formulations, reported with much high annual trade value, and are among widely traded RPD’s from India (Table 2). The literature review revealed that several closely related species have similar names. Similarity and confusion in local or trade names of many species are often reported with adulteration problems. For example, *C. procera* and *C. gigantea* have the same ayurvedic name, *i.e*., *Alarka*. Similarly, the roots of three selected plants, viz., *C. dubia*, *H. indicus,* and *I. frutescens* are known as “Sariva” in Sanskrit. Due to the similar common name, the official part of true ‘Sariva’ (*H. indicus*) is known to be adulterated by the other two plants of the same common name.

**Table 2:** Details of the studied root drug samples (RDS) of the plant species belonging to the family Apocynaceae used in ISM

Botanical name	Subfamily	Accession No. (RRLH-CDR-)	Place of Collection	Synonyms (TPL, 2013)	Local/ Trade names	Ayurvedic name (API, 2001; Khare, 2007)	Estimated annual trade	Adulterants/ Potential confounding material
*Asclepias curassavica *L.	Asclepiadoideae	3806	Pune, Maharashtra	*Asclepias cubensis *Wender., *Asclepias curassavica* var. *concolor* Krug & Urb., *Asclepias nivea* var. *curassavica* (L.) Kuntze	Curassavian Swallow-wort, Kaakanaasikaa, Kaakatundi	Kaakanaasikaa	< 10 MT (NMPB, 2020)	*Leptadenia reticulata* (Retz.) Wight & Arn. (Ramesh et al. 2014**)**
*Calotropis gigantea* (L.) Dryand.	Asclepiadoideae	2122	Bhopal, Madhya Pradesh	*Asclepias gigantea *L., *Calotropis gigantea* (L.) R. Br. ex Schult., *Madorius giganteus* (L.) Kuntze	Madar, Giant Milk-weed, Erukkin veru, Aak	Arka, Alarka, Raajaarka, Shvetaarka, Vasuka, Mandaar, Bhaasvanmuula, Dinesh, Prabhaakara, Ravi, Bhaanu, Tapana	50–100 MT (NMPB, 2020)	*C. procera* (Aiton) Dryand. (Sarin 1996)
*Calotropis procera* (Aiton) Dryand.	Asclepiadoideae	2805	Bhopal, Madhya Pradesh	*Asclepias procera* Aiton, *Calotropis gigantea* var. *procera* (Aiton) P.T.Li, *Calotropis heterophylla* Wall. ex Wight	Swallow-Wart, Milk Weed, King’s Crown, Akada Phool	Alarka, Surya, Suuryaahvya, Vikirna, Vasuka, Tapana, Tuulaphala, Kshirparna, Arkaparna, Aasphota	50–100 MT (NMPB, 2020)	*C. gigantea* (L.) Dryand. (Sarin 1996)
*Carissa carandas* L.	Rauvolfioideae	2198	Bhopal, Madhya Pradesh	*Arduina carandas* (L.) Baill., *Carissa salicina* Lam., *Capparis carandas* (L.) Burm.f	Christ’s Thorn, Bengal, Currant	Karinkaara, Karamarda, Krishnapaakphal, Kshirphena, Sushena	NA	NA
*Carissa spinarum* L.	Rauvolfioideae	2966	Lucknow, Uttar Pradesh	*Carissa abyssinica* R. Br., *Carissa* *carandas* var. *congesta* (Wight) Bedd., *Carissa opaca* Stapf ex Haines	Jangali Karondaa, Garnaa	Karamardikaa	NA	*Carissa* *paucinervia* A.DC. (Khare 2007)
*Catharanthus roseus* (L.) G. Don	Rauvolfioideae	1650	Bhopal, Madhya Pradesh	*Catharanthus roseus* var. *albus* G. Don, *Lachnea rosea* (L.) Rchb., *Vinca rosea* L.	Sadaabahaar, Nayantaaraa, Nityakalyaani, Madagascar Periwinkle, Vinca	NA	200–500 MT (NMPB, 2020)	*Solanum melongena* L.*, Lycopersicon esculentum* Mill., *Ocimum tenuiflorum* L. (Ganie et al. 2015; Nithaniyal et al. 2016)
*Cryptolepis dubia* (Burm.f.) M.R.Almeida	Periplocoideae	4088	Jammu, Jammu & Kashmir	*Cryptolepis buchananii* Roem. & Schult., *Cryptolepis reticulata* (Roth) Wall. ex Steud., *Nerium reticulatum* Roxb.	Indian Sarsaparilla, Karantaa, Anantamuula, Medaksinghi, Krsnasariva, Sveta sariva	Krsnasariva, Krishna Saarivaa, Jambupatraa Saarivaa, Arantaa, Shyamalataa, Shyaama, Gopi, Gopavadhu, Kaalghatika	100–150 MT (NMPB, 2020)	*Periploca calophylla* (Wight) Falc., *Ichnocarpus frutescens* (L.) W.T.Aiton, *Decalepis hamiltonii* Wight & Arn., *Hemidesmus indicus* (L.) R. Br. ex Schult. (Khare 2007; Jeewandara et al. 2017; Sarin 1996)
*Hemidesmus indicus* (L.) R. Br. ex Schult.	Periplocoideae	471	Jammu, Jammu & Kashmir	*Periploca indica* L.	Indian Sarsaparilla, Anatmool, Sariwa, Sveta sariva	Shveta Saarivaa, Anantmuula, Gopi, Gopaa, Gopakanyaa, Gopavalli, Gopasutaa, Krishodari, Sphotaa, Utpalsaarivaa, Kapuuri, Dugdhgarbhaa	500–1000 MT (NMPB, 2020)	*Decalepis hamiltonii* Wight & Arn., *Periploca calophylla* (Wight) Falc., *Krameria triandra* Ruiz & Pav., *Saccolabium papillosum* Lindl., *Smilax aspera* L., *Smilax ovalifolia* Roxb. ex D.Don, *Ichnocarpus frutescens* (L.) W.T.Aiton, *Cryptolepis dubia* (Burm.f.) M.R.Almeida (Sarin, 1996; Khare 2007; Jeewandara et al. 2017)
*Holarrhena pubescens* Wall. ex G.Don	Apocynoideae	2514	Kolkata, West Bengal	*Holarrhena antidysenterica* (Roth) Wall. ex A.DC., *Holarrhena codaga* G.Don., *Holarrhena glabra* Klotzsch	Easter tree, Ivory tree, Tellicherry Bark	Indrayava, Kutaja, Girimallikaa, Kaalinga, Kalingaka, Indravriksha, Shakra, Vatsa, Vatsaka, Shakraahvya	1000–2000 MT (NMPB, 2020)	*Ailanthus excelsa* Roxb. (Khare 2007)
*Ichnocarpus frutescens* (L.) W.T.Aiton	Apocynoideae	1912	Lucknow, Uttar Pradesh	*Apocynum frutescens* L., *Ichnocarpus affinis* (Roem. & Schult.) K.Schum., *Tabernaemontana* *parviflora* Poir.	Black Creeper	Gopavalli, Krishna, Saarivaa, Krishna–muuli, Shyaamalataa	NA	*Cryptolepis dubia* (Burm.f.) M.R.Almeida, *Decalepis hamiltonii* Wight & Arn., *Hemidesmus indicus* (L.) R. Br. ex Schult. (Khare 2007; Jeewandara et al. 2017)
*Marsdenia tenacissima* (Roxb.) Moon	Asclepiadoideae	3232	Gwalior, Madhya Pradesh	*Gymnema tenacissimum* (Roxb.) Spreng., *Marsdenia tenacissima* Wight & Arn., *Asclepias tenacissima* Roxb.	Maruaa-bel, Khaarchu, Nishod, Sufed Murva	Murva, Muurvaa	10–20 MT (NMPB, 2020)	*Operculina turpethum* (Linn.), *Ipomoea turpethum* R. Br. (Khare 2007; Kolhe et al. 2014)
*Nerium oleander* L.	Apocynoideae	541	Bhopal, Madhya Pradesh	*Nerium indicum* Mill., *Nerium japonicum* Gentil., *Nerium latifolium* Mill.	Indian oleander, White Oleander, Oleander, Kaner, Karavira	Karavira, Viraka, Ashvamaaraka, Hayamaaraka, Gauripushpa, Divyapushpa, Shatakumbha, Siddhapushpa, Raktapushpa, Raktaprasava, Ravipriya	< 10 MT (NMPB, 2020)	The root bark is known to be adulterated and substituted by stem bark (Sarin 1996)
*Rauvolfia serpentina* (L.) Benth. ex Kurz	Rauvolfioideae	413	Jammu, Jammu & Kashmir	*Rauvolfia obversa* (Miq.) Baill., *Rauvolfia trifoliata* (Gaertn.) Baill., *Ophioxylon album* Gaertn	Rauvolfia root, Serpentina root, Indian Snakeroot	Sarpagandha	200–500 MT (ENVIS, 2020)	*Rauvolfia tetraphylla* L., *Rauvolfia densiflora* (Wall.) Benth. ex Hook.f.*, Rauvolfia micrantha* Hook. f., *Ophiorrhiza mungos* L., *Clerodendrum* species, *Tabernaemontana divaricata* (L.) R.Br. ex Roem. & Schult., *Rauvolfia beddomei* Hook.f., *Rauvolfia verticillata* (Lour.) Baill. (Sarin 1996; ENVIS 2020)
*Tabernaemontana divaricata* (L.) R.Br. ex Roem. & Schult.	Rauvolfioideae	2078	Bhopal, Madhya Pradesh	*Nerium divaricatum* L., *Tabernaemontana coronaria* (Jacq.) Willd., *Vinca* *alba* Noronha	East Indian Rosebay, Chandni	Tagar, Nandivriksha, Nandi Pushpa	< 10 MT (NMPB, 2020)	*Valeriana hardwickii* Wall., *Cedrela toona* Roxb. ex Rottler (Khare 2007)

*MT* Metric Tonnes, *NA* not available

### Botanical characterisation of root samples

**Fig. 1:**
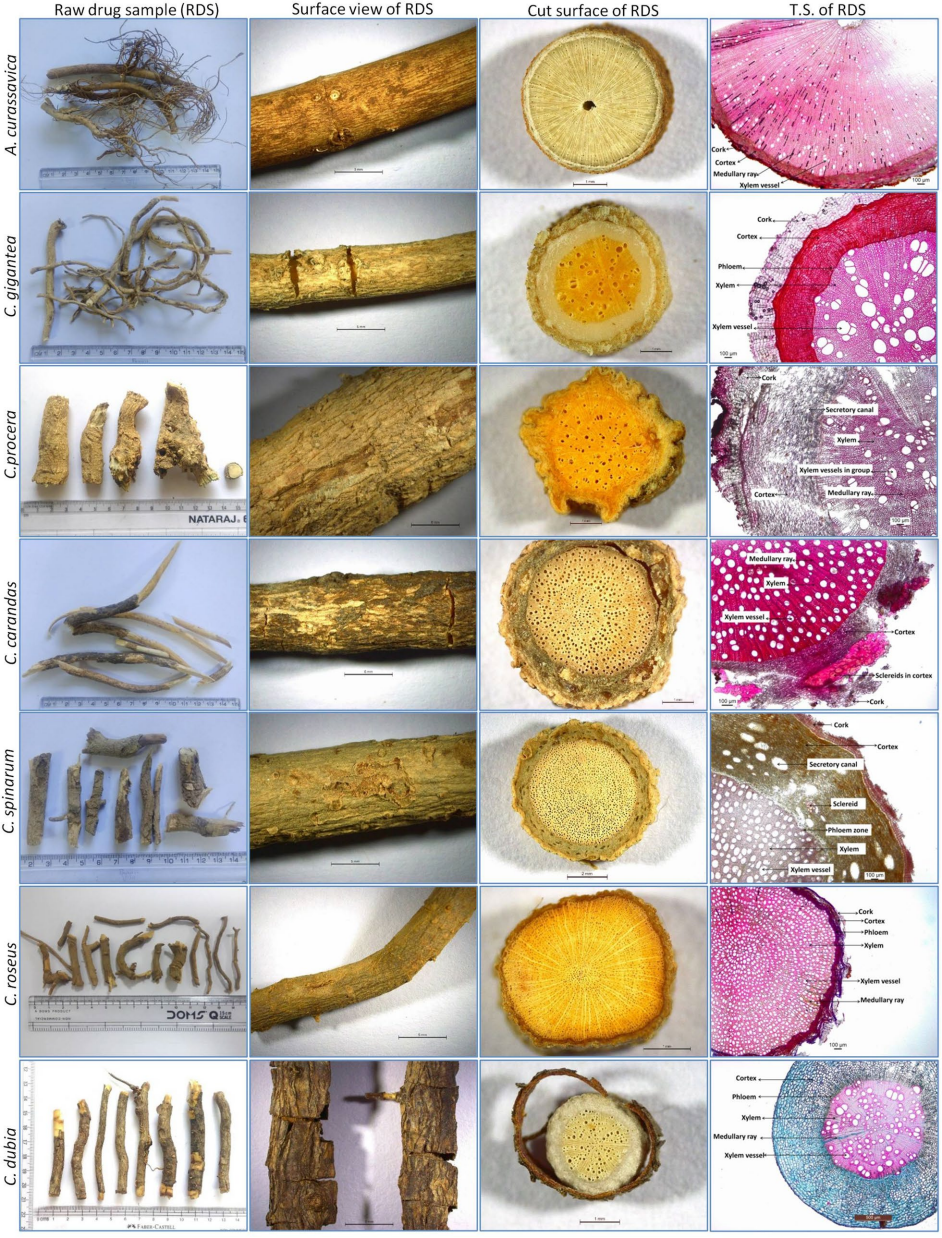
Morpho-anatomical images of Raw Drug Sample (RDS) of the studied seven roots of the family Apocynaceae used in ISM (*A. curassavica* to *C. dubia*)

**Fig. 2:**
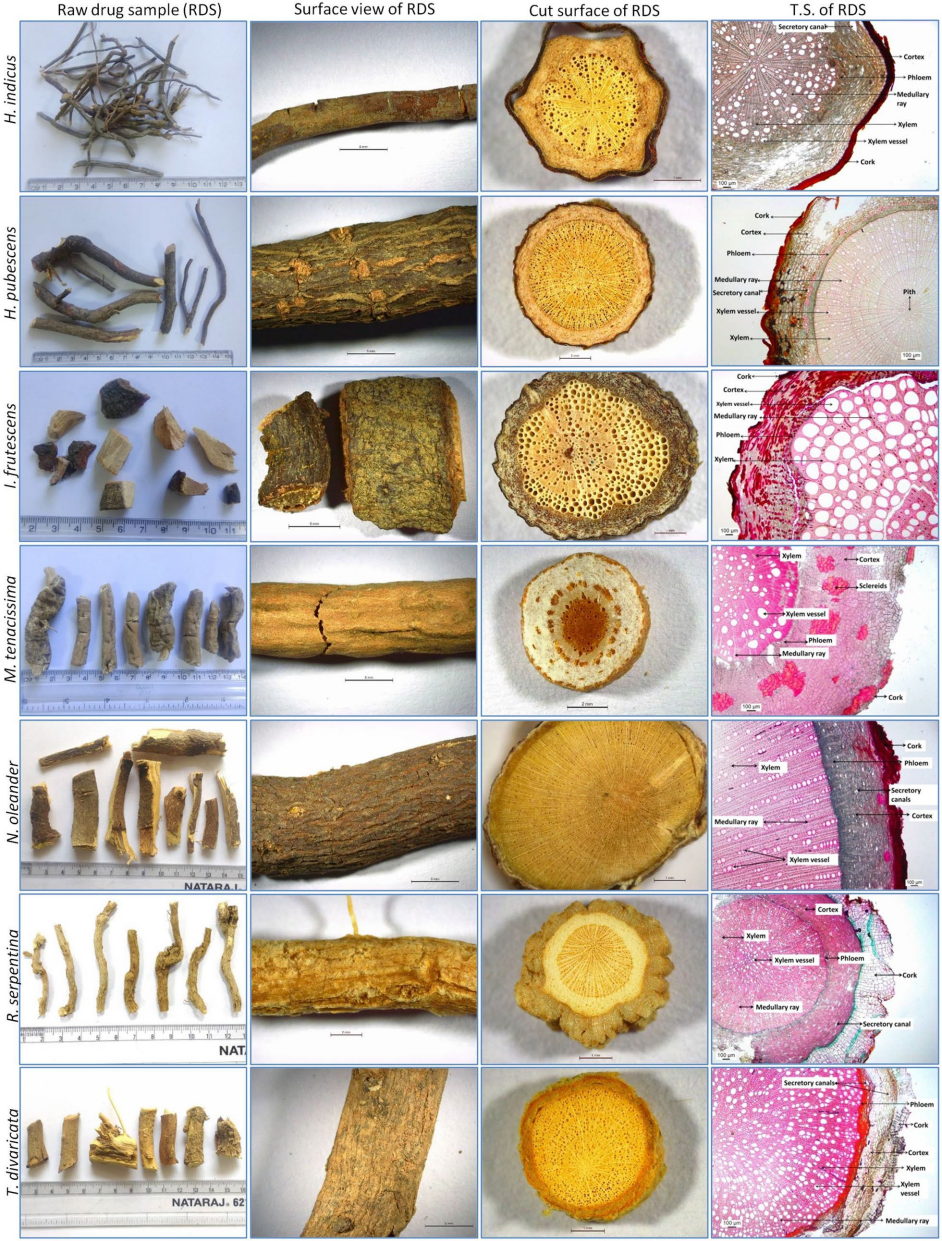
Morpho-anatomical images of Raw Drug Sample (RDS) of the studied seven roots of the family Apocynaceae used in ISM (*H. indicus* to *T. divaricata*)

**Fig. 3:**
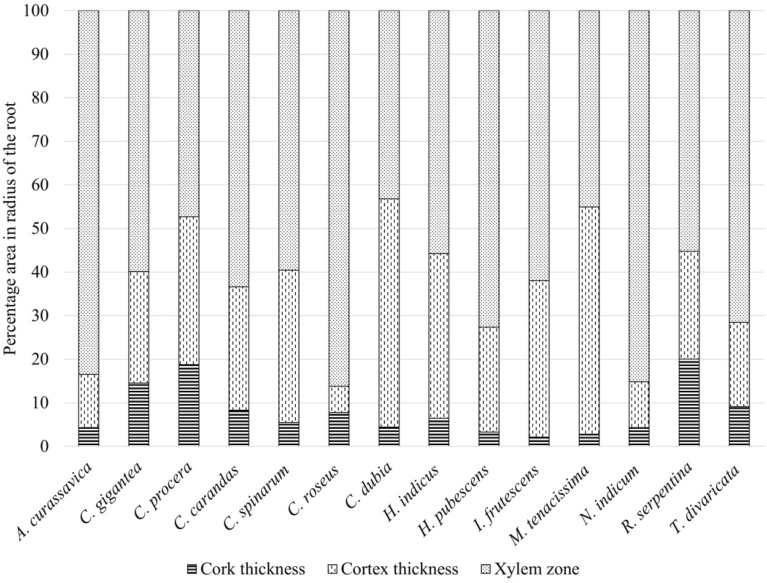
Relative area of cork, cortex, and xylem zones based on the radius of the studied samples of the 14 species of family Apocynaceae used in ISM

In the comparative morphological study, sample appearance, surface, and cut root appearance were studied. Comparative morphological characteristics of the studied RDS are shown in Figs. 1, 2. Root drug samples of studied species appeared similar in physical appearance and morphological features, while some surface and cut root features were characteristic. RDS of most species appeared elongated or cylindrical, less branched, twisted, or bent, but *A. curassavica* was observed with secondary and tertiary fibrous branches. The root surface of most drug samples was rough with wrinkles (in *C. procera*, *C. dubia*, *M. tenacissima*, *T. divaricata*), cracks (in *C.* *gigantea*, *C. carandas*, *C. dubia*, *H. pubescens*, *M. tenacissima*, *N. oleander*), some with a powdery mass on scraping (in *C. gigantea*, *C. procera*, *C. spinarum*, *R. serpentina*, *T. divaricata*), and sloughed off bark (in *C. carandas*, *C. dubia*, *H. indicus*), and nodule like protuberances (in *C. spinarum*). Some species were with smooth root surfaces (*A. curassavica*, *C. roseus*, *M. tenacissima*). Root surface was of variable color such as greenish (in *A. curassavica*), light cream (in *C.* *gigantea*, *R. serpentina*), buff-colored (in *C. procera*, *M. tenacissima*, *T. divaricata*), dark brown (in *C. dubia*, *H. indicus*, *H. pubescens*, *I. frutescens*), dark brown with light patches (in *C. carandas*), brown (in *N. indicum*), light brown (in *C. spinarum*) to light green (in *C. roseus*). Cut root surface was circular in most species while irregular outline in *C. procera*, *H. indicus*, *I. frutescens*, *R. serpentina* and circular to oval in *C. dubia*. The bark and woody region showed variability in thickness (Fig. 1, 2, Fig. 3). Woody region showed variation in pore size and characteristic pattern of pores. Pores in most species were of varying size, having well-distinguished large pores, while some had very small pores (in *A. curassavica*, *C. roseus*, *H. pubescens*, *R. serpentina*) (Table 3).

**Table 3:** Comparative morphological characteristics of the studied RDS of family Apocynaceae used in ISM

Plant name	Raw root drug sample appearance (Figs. 1, 2)	Surface morphological characters (Figs. 1, 2)	Cut root surface characters (Figs. 1, 2)
*A. curassavica*	Slender, elongated, with several secondary and tertiary adventitious, fibrous roots	Samples smooth with no apparent wrinkles or furrows, greenish, root scars at some points. Few samples twisted with bends and secondary branches	Nearly circular in outline with a thin greenish-brown patch of cork zone followed by a comparatively light small zone. The primary central zone comprises a light brown woody part with numerous randomly distributed pores of very small size and several creamish rays emerging from the central region. A pith represents a small central hollow part
*C. gigantea*	Mostly thick roots with some comparatively thinner roots	Surface rough, light cream, with longitudinal fissures and transverse cracks. A powdery mass formed on the scrapping of bark	Nearly circular in outline, cork region light green, inner cortex region creamy pale. Central woody part nearly circular, pale with randomly scattered pores of varying size
*C. procera*	Mostly thick roots with some comparatively thinner roots	Surface rough, wrinkled, buff-colored, cylindrical, or irregular shaped. A powdery mass formed on the scrapping of bark	Irregular in outline, pale or light brown, thick bark protruded on sides, inner woody part pale having randomly scattered pores of varying size
*C. carandas*	Woody, hard, slender, less branched	Surface rough, dark brown with light patches in between. Bark with transverse cracks may be sloughed off from the woody part	Nearly circular in outline with the irregular outer surface, outer few patches light. Reddish-brown inner bark with less differentiation between cork and cortex, central woody part comprised of nearly uniformly distributed pores of varying size showing circular and spoke like arrangement. Bark may separate from the woody part
*C. spinarum*	Woody, hard, slender, and branched at some points	Surface light brown with wrinkles and nodule-like protuberances. A powdery mass formed on the scrapping of bark	Nearly circular with irregular bark outline. Bark light brown having several reddish-brown spots and a comparatively light central large woody region with several uniformly distributed pores of varying sizes
*C. roseus*	Varying thickness branched at some points with the light green surface	Surface light green, compact, and nearly smooth with no noticeable wrinkles or cracks	It may or may not be circular in outline. Outer surface corky and greenish, central part large, woody, pale with several small-sized evenly distributed pores traversed by spoke like rays
*C. dubia*	Elongated cylindrical, dark brown, branched at few points	Surface dark brown, wrinkled with several transverse cracks. Bark separated from the woody portion at several places	Cut root with sloughed off dark-colored scaly bark, inner cortex region creamish white, and central woody part with randomly scattered pores of varying size in the spoke-like arrangement. Some spoke like rays appear emerging from the center part
*H. indicus*	Elongated and of varying thickness	Surface rough, dark brown, or dark buff-colored, with transverse cracks. Bark separated from the woody portion at some places	Cut root surface irregular in outline with few small protrusions at some points, outer region thin, dark brown, maybe separated at few points from cortex part of main bark, cortex part light pale, central woody part appears nearly circular in outline with randomly scattered pores of varying size. Spoke-like rays emerging from the central part were also observed
*H. pubescens*	Cylindrical, thick, and long with few branches	Surface rough dark brown with numerous small longitudinal and transverse cracks	Circular in outline, the thin outer region is dark brown, inner light pale green zone with few small brown patches. Central woody part with small-sized pores of uniform distribution spoke like thin rays emerging from the central part. The root may show annular ring-like markings
*I. frutescens*	Dark brown woody pieces	Surface dark brown, rough with thick bark	Irregular in outline with a thin dark brown outer cork layer, inner cortex region thick with several small transversally flattened, dark brown, and reddish-brown patches. Central woody part irregular in outline with several uniformly distributed pores of varying size, arranged in a nearly circular pattern
*M. tenacissima*	Thick, cylindrical, less branched	Transversely cracked bark, surface deformed, appeared wrinkled with a nearly soft or smooth texture and buff-colored surface	Nearly circular in outline with outer thin, light green patches and inner large creamy white cortex zone with several golden-red spots. The central woody zone formed a comparatively small region than bark. Woody part relatively dark, comprised of several scattered pores of varying sizes
*N. oleander*	Elongated, thick with the dark brown surface	Less branched with brownish bark having small longitudinal fissures	Nearly circular or oval-shaped, bark thin with dark brown outer cork region, cortex region appears greenish-brown. Centre occupied by large woody part with a dilated center point having unevenly distributed small-sized pores of varying size
*R. serpentina*	Elongated, slender, thin, less branched, and irregular in shape	Surface rough and creamish pale. A powdery mass formed on the scrapping of bark	Irregular in outline with bark surface protuberances, outer cork region buff or light pale and inner cortex region formed a nearly circular, light cream band. The central part is the woody region with small-sized, uniformly distributed pores traversed by spoke-like rays emerging from the center (maybe dilated to one side)
*T. divaricata*	Long, slender, less branched, and of varying thickness	Surface buff-colored and rough, longitudinally wrinkled. Little powdery mass formed on scrapping of bark	Nearly circular in outline with greenish-brown bark region and large central pale woody region with uniformly distributed pores of varying size, and spoke like rays emerging from the center. The central part of the wood may appear dilated to one end

Xylem vessel arrangement varied from solitary (in *C. carandas*, *C. spinarum*), grouped (in *C. gigantea*, *C. procera*), linear (in *N. oleander*), grouped and linear (in *H. pubescens*), solitary and grouped (in *C. roseus*, *C. dubia*, *H. indicus*, *I. frutescens*, *M. tenacissima*), solitary and linear (in *A. curassavica*), solitary, linear and grouped (in *R. serpentina*, *T. divaricata*). Medullary rays were less distinct (in *A. curassavica*, *C. procera*, *C. roseus*), distinct (in *C. carandas*, *C. spinarum*, *H. pubescens*, *M. tenacissima*, *R. serpentina* and *T. divaricata*), narrow (in *C. gigantea*, *C. dubia*, *H. indicus*, *I. frutescens*, *N. oleander*). Pith was present in some species (*A. curassavica*, *H. pubescens*, *I. frutescens*).

Powder microscopic study of most species showed cork cell fragments, parenchyma cell fragments, sclereid fragments, coloured fragments, prismatic crystals, rosette crystals, starch grains, xylem vessel fragments. However, variability was observed in cells and cell contents such as starch grains and crystals. Starch grains of most species were solitary to compound (3–4 units), some up to 2 units (*T. divaricata*), and some up to 9 units (*M. tenacissima*). The shape of starch grains varied from spherical (in *C. dubia*), oval to spherical (*C. gigantea*, *C. procera*, *C. carandas*, *C. spinarum*, *C. roseus*, *I. frutescens*, *M. tenacissima*, *T. divaricata*), oval to elongated (*H. indicus*, *H. pubescens*, *N. oleander*), to more than one shape (*A. curassavica* and *R. serpentina*). Among studied species, prismatic crystals were present in all root samples except *M. tenacissima*. Apart from these, rosette crystals were also observed in some species (*A. curassavica*, *C. procera*, *H. pubescens*, *M. tenacissima*, *N. oleander,* and *T. divaricata*). The size of starch grains and prismatic crystals are provided in Table 4.

**Table 4:** Comparative characteristics of the root powder of the studied RDS of family Apocynaceae used in ISM (Additional file 1: Figs. S1, S2)

Plant name	Organoleptic characters				Type of xylem vessel fragments	Starch grains			Crystals			Other microscopic structures observed in root powder
	Colour	Odour	Taste	Texture		Type	Size		Type	Prismatic crystal size		
							Length ± SE (range) µm	Breadth ± SE (range) µm		Length ± SE (range) µm	Breadth ± SE (range) µm	
*A. curassavica*	Creamish brown	Bitter or pungent characteristic	Bitter	Sandy or granular	Simple pitted	Few compound starch grains of variable shapes, singly or in a group of up to 4 units	10.85 ± 1.16ab (5.53–19.38)	9.58 ± 1.11ab (5.55–17.42)	Rosettes and prismatic	25.07 ± 2.80 cd (10.34–38.32)	16.90 ± 2.14b (6.41–26.95)	Few golden–brown fragments Few sclerenchyma cells fragments
*C. gigantea*	Creamish white	Faint characteristic	Bitter	Nearly soft	Simple pitted	Abundant oval to circular compound starch grains singly or in a group of up to 4 units	14.14 ± 1.43a (8.87–22.55)	12.74 ± 1.30a (7.08–20.60)	Polygonal prismatic	22.40 ± 2.36 cd (12.44–36.59)	18.25 ± 1.99b (8.52–28.43)	Cork cells fragments Parenchyma cells fragments Few coloured fragments Few sclerenchyma cells fragments
*C. procera*	Light brown or pale with faintly pale fragments	Faint characteristic	No taste	Flaky, smooth	Border pitted	Abundant circular to oval compound starch grains singly or in a group of 3 units	12.18 ± 1.21ab (5.34–17.17)	9.22 ± 0.82ab (4.04–12.52)	Numerous rosette and prismatic, few acicular	39.01 ± 3.49ab (24.58–63.16)	30.71 ± 3.40a (19.20–58.19)	Cork cells fragments Few reddish-brown fragments
*C. carandas*	Creamish white with a slight pale tinge	Soil like faint characteristic	No taste	Flaky, granular	Border pitted	Numerous circular to oval compound starch grains singly or up to 4 or more units	9.43 ± 1.06ab (4.75–15.33)	8.20 ± 0.74b (4.34–12.89)	Prismatic crystals of varying shapes	22.26 ± 1.80 cd (11.57–28.14)	18.19 ± 2.08b (8.13–26.18)	Reddish-brown fragments Numerous sclereids fragments
*C. spinarum*	Creamish white with a slight pale tinge	Soil like faint characteristic	No taste	Flaky, gritty, granular	Border pitted	Numerous circular to oval compound starch grains singly or grouped up to 3 or more units	11.71 ± 1.22ab (6.02–17.34)	9.65 ± 1.02ab (3.95–15.15)	Prismatic crystals of varying shapes	18.42 ± 2.30 cd (12.09–36.94)	11.92 ± 1.35b (7.35–22.42)	Reddish-brown fragments Numerous sclereids fragments
*C. roseus*	Pale with few light brown fragments	Faint characteristic	Unpleasant bitter	Flaky, sandy	Simple pitted	Few oval to circular starch grains mostly singly	10.19 ± 1.09ab (5.20–14.55)	8.50 ± 0.90ab (4.50–12.60)	Prismatic crystals of varying shapes	46.03 ± 4.08a (29.44–67.81)	33.06 ± 3.66a (20.58–54.01)	Few cork cells fragments Several golden-brown fragments Few sclereids fragments
*C. dubia*	Dark brown with few light brown fragments	No odor	No taste	Gritty, flaky	Border pitted	Abundant circular compound starch grains singly or up to 4 units	10.42 ± 1.19ab (4.89–18.34)	9.60 ± 1.09ab (4.61–16.85)	Prismatic crystals mostly of the rectangular shape	13.52 ± 1.54 cd (8.12–25.26)	9.97 ± 0.64bc (7.61–13.35)	Few cork cells fragments Parenchyma cells fragments with starch grains Few golden-brown fragments Few sclereids fragments
*H. indicus*	Light brown with several dark brown fragments	No odor	No taste	Granular, smooth	Border pitted	Abundant oval to elongated compound starch grains singly or up to 3 units	11.46 ± 0.69ab (8.87–16.27)	8.77 ± 0.59b (6.42–12.36)	Prismatic crystals of varying shapes	21.95 ± 2.69 cd (13.87–34.69)	13.68 ± 1.11b (10.33–22.09)	Several reddish-brown fragments Few sclerenchyma cell fragments
*H. pubescens*	Pale with few dark brown fragments	Faint characteristic	Faint bitter	Rough, gritty	Border pitted	Numerous oval to elongated, compound starch grains singly or up to 3 units	9.02 ± 0.66ab (4.87–12.04)	6.97 ± 0.41ab (4.49–9.33)	Rosette and prismatic crystals of varying shapes	16.87 ± 1.46 cd (9.62–25.10)	15.06 ± 1.51b (7.92–22.86)	Few deep-reddish fragments Sclereids fragments
*I. frutescens*	Medium brown with few dark brown fragments	No odor	No taste	Rough, gritty	Border pitted	Numerous oval to circular, compound starch grains mostly singly	11.43 ± 0.62ab (7.21–13.76)	8.81 ± 0.57b (5.28–10.88)	Prismatic crystals of varying shapes	22.28 ± 1.59 cd (15.52–30.43)	16.28 ± 1.34b (11.74–24.82)	Several deep-reddish fragments Sclereids fragments
*M. tenacissima*	Creamish white	Faint pleasant characteristic	Faint bitter	Smooth (flour-like) with slight granular touch	Few with simple pits	Few oval to circular, abundant compound starch grains singly or grouped up to 9 units	9.08 ± 1.15ab (5.28–16.63)	7.81 ± 0.94b (4.46–13.52)	Rosette crystals only, prismatic crystals not observed	-	-	Numerous sclereids fragments Colored fragments not observed
*N. oleander*	Light brown	Faint characteristic	Bitter	Flaky texture	Simple pitted	Few oval to elongated, compound starch grains singly or up to 3 units	7.10 ± 0.73b (3.73–10.48)	5.83 ± 0.47b (3.34–7.75)	Rosette and prismatic crystals of varying shapes	28.37 ± 4.93bc (12.81–58.41)	17.87 ± 4.64b (7.96–52.09)	Few deep-reddish fragments Few sclereids fragments
*R. serpentina*	Creamish white with few faintly pale fragments	Characteristic	Bitter	Flaky, smooth	Simple pitted	Abundant compound starch grains of various shapes singly or up to 4 or more units	11.88 ± 1.55ab (5.37–19.15)	10.08 ± 1.26ab (4.74–15.81)	Few prismatic crystals of varying shapes	22.25 ± 1.77 cd (15.50–35.01)	12.34 ± 1.48b (8.11–23.04)	Cork cells fragments Few coloured fragments Few sclereids fragments
*T. divaricata*	Pale brown with few dark brown fragments	Slightly pleasant	No taste	Rough, gritty	Simple pitted	Oval to circular starch grains singly or in groups up to 2 units	8.56 ± 0.53b (6.21–10.79)	7.91 ± 0.47b (6.18–9.95)	Rosette and prismatic crystals are mostly rectangular	22.07 ± 2.77 cd (9.05–34.52)	15.66 ± 2.40b (5.61–30.25)	Few golden-brown fragments Few sclereids fragments

S.E. = Standard Error

One-way ANOVA’s were carried out separately for each quantitative character to figure out the differences among different species. The same letters after values in a column denote a lack of statistically significant differences, according to Tukey’s post hoc test (p < 0.05)

**Fig. 4:**
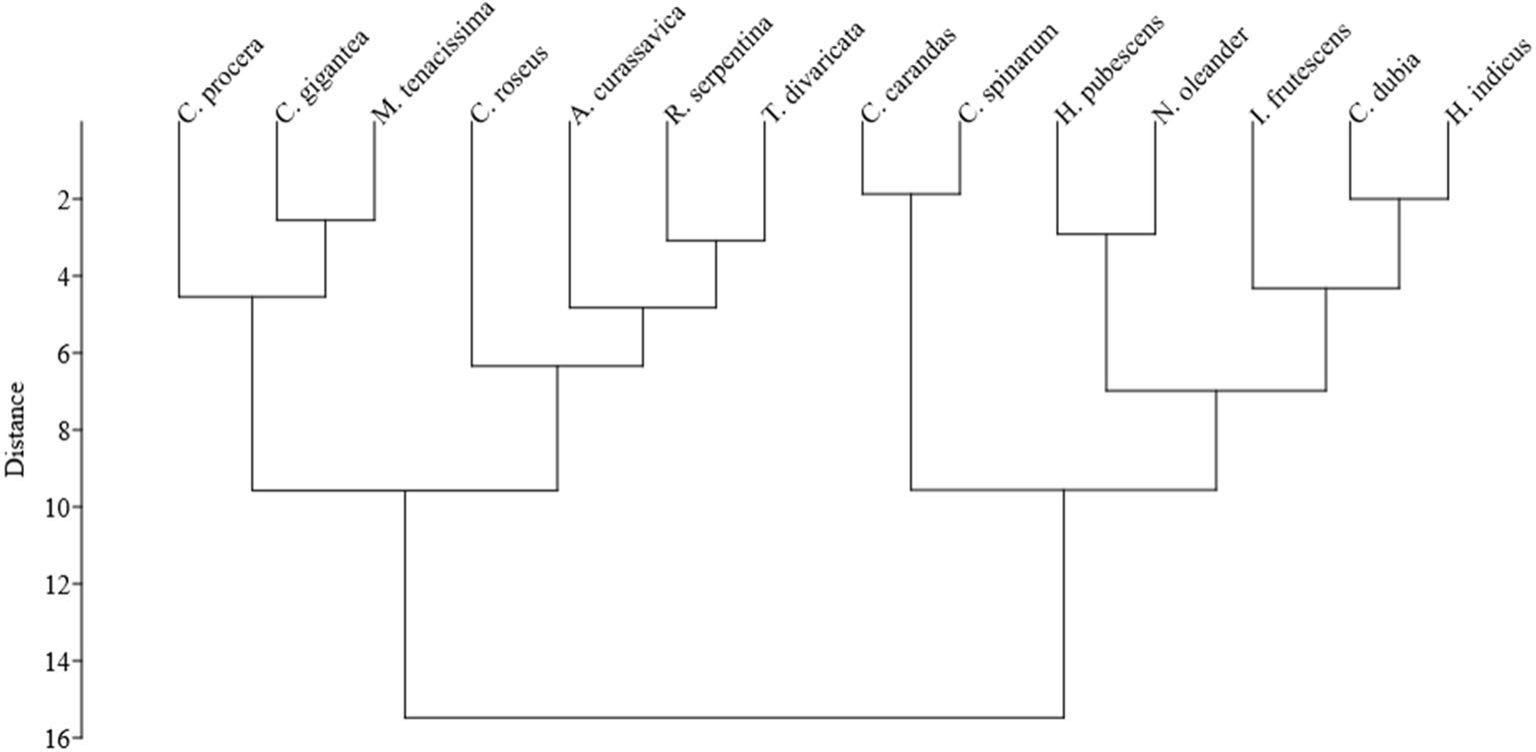
Cluster analysis dendrogram showing interrelationships between 14 species of family Apocynaceae used in ISM based on 33 morphological, anatomical, and powder characters of the RDS

**Fig. 5:**
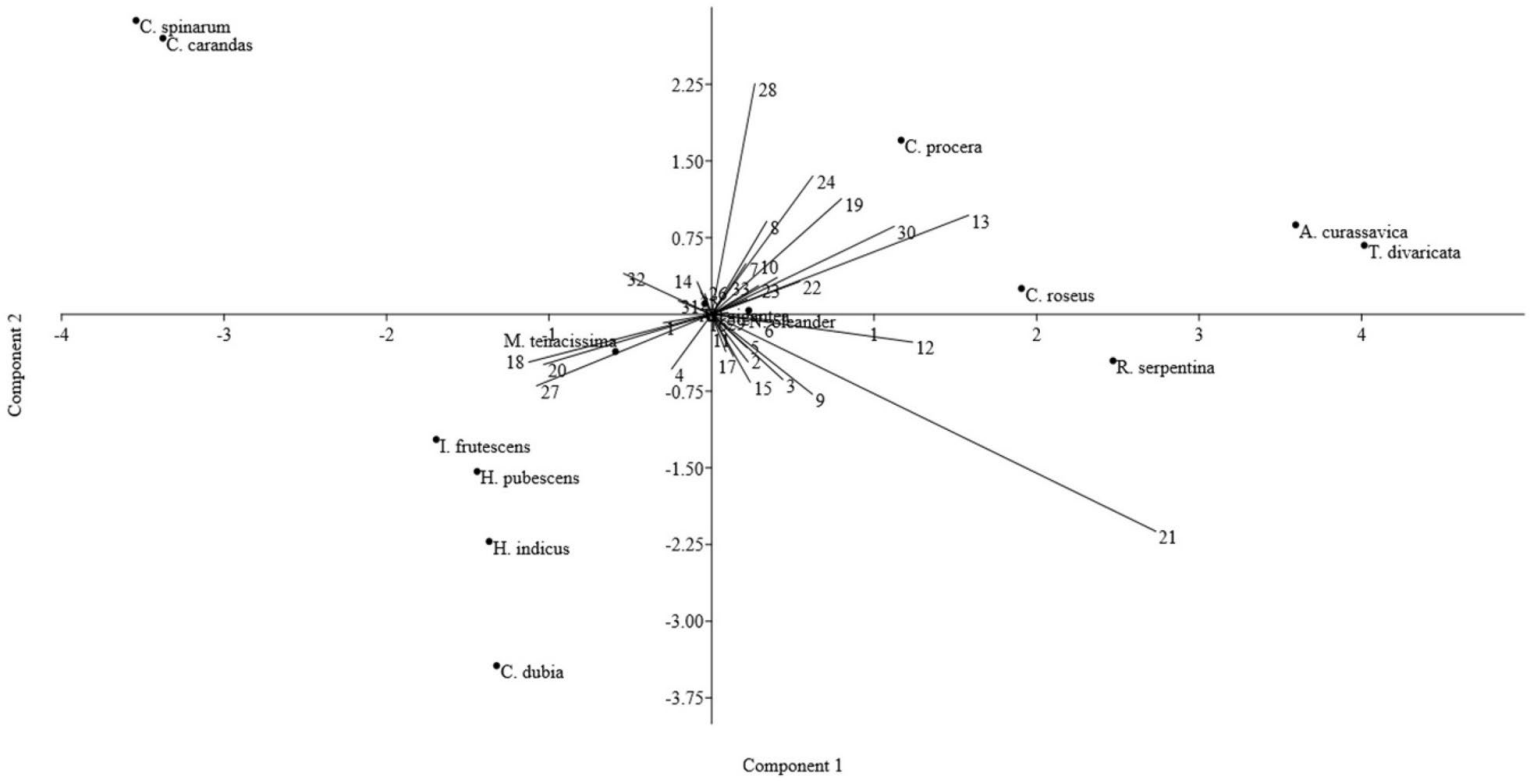
Scatter plot diagram of PCA showing important RDS characters for the distinction of 14 species of family Apocynaceae used in ISM

In the current study, some characters were shared in studied species, while some features were also observed as characteristics useful in species distinction. Statistical analysis of studied botanical characters by the mean–variance analysis (Tables 4, 5), PCA, and Cluster analysis was observed to resolve the complexity in species distinction and identification of significant characters. The cluster analysis results are represented in a dendrogram, which shows closely related species’ grouping (Fig. 4). A Scatter plot diagram of PC1 versus PC2 showed significant characters with taxonomic value in the grouping and distinction of various species (Fig. 5). PCA analysis showed that the first three components accounted for nearly 64% of the total variance (30.49%, 16.96%, and 16.58%, respectively). According to the first three PCA’s, the following characters including clustering of xylem vessel, the shape of starch grains, pores in woody part (size, arrangement, and distribution in the cut root), the width of medullary rays, the thickness of dominating tissues in T.S. (cork, cortex and xylem zones), cork lignification and cork colour in the cut root, type of crystals, starch distribution test in T.S. of the root, surface characters of raw drug sample such as texture, wrinkles, fissures, etc., were observed as major contributors in the whole variation. Also, mean–variance analysis of quantitative features revealed the mean thickness of tissue zones in cross-section, vessel lumen diameter and number of vessels per square area, mean size of starch grains, and prismatic crystals as major characters in species distinction.

**Table 5:** Comparative anatomical characteristics of the root of the studied RDS of family Apocynaceae used in ISM (Fig. 1–2)

Plant name	Mean radius of studied root samples ± SE (range) [n = 5] µm	Cork (outer bark)		Inner bark					Xylem				Pith	Medullary rays	Iodine test of dry T.S. showing an abundance of starch in
		General characters	Mean thickness ± SE (range) µm	Cortex (C)			Phloem (P)	C + P, Mean thickness ± SE (range) µm	General characters	Xylem zone mean length ± SE (range) µm	Vessel lumen mean diameter ± SE (range) µm	No. of vessels per 10^6^ µm^2^ ± SE (range)			
				General characters	Secretory canals										
					Length ± SE (range) µm	Breadth ± SE (range) µm									
*A. curassavica*	2581.14 ± 12.79 (2517.75–2623.74)	Outermost broken parenchymatous zone with lignified outer cork cells	107.23 ± 6.25efg (80.16–138.02)	Ten to twelve layered parenchymatous zone. Transversally elongated cells filled with starch grains Small patches of sclereids Few rosettes, prismatic calcium oxalate crystals are present Small elongated or deformed secretory canals are present	47.55 ± 4.69b (21.44–68.25)	23.57 ± 2.72c (13.46–40.45)	Small and dark-colored zone	303.17 ± 12.05 h (272.71–399.20)	Significant central part of T.S. of the root formed of xylem fibrillar part Small-sized xylem vessels, being randomly present, forming linear and solitary arrangement	2073.55 ± 5.61b (2047.30–2104.19)	32.42 ± 3.56d (18.17–53.97)	62.00 ± 1.07ef (57–68)	Present	Less distinct	Cortex
*C. gigantea*	2207.46 ± 86.08 (1874.21–2608.47)	Outermost zone of parenchymatous, rectangular cells	332.12 ± 13.25bc (237.28–401.12)	Wide cortex with up to 25 layers of parenchyma cells filled with starch grains Very small circular secretory canals are present	38.85 ± 3.15b (22.44–53.77)	25.13 ± 2.07c (15.47–36.59)	Small, less distinct	583.01 ± 20.65def (466.75–690.74)	The central part with major xylem fiber and parenchyma, separated by medullary rays Vessels scattered, of variable lumen size, present in groups of 2–6	1366.48 ± 45.08efg (1186.60–1565.87)	134.54 ± 24.21a (30.15–275.78)	22.80 ± 0.48i (20–25)	Absent	Narrow, distinct	Cortex and medullary rays
*C. procera*	2876.81 ± 21.20 (2768.10–2978.51)	Outermost zone of broken cell layers with outer few cell layers lignified, and inner cell layers parenchymatous	545.86 ± 66.45a (333.31–908.04)	Thick cortex with around 30 cells layered parenchymatous zone Rosette crystals are present Small secretory canals are present	68.16 ± 3.65b (47.84–81.65)	42.18 ± 2.28c (33.12–58.55)	Small, less distinct zone	987.79 ± 32.39b (742.61–1095.66)	The central part with the significant part formed of xylem fiber and parenchyma Vessels scattered, of variable lumen size, present in groups of 2–5	1374.59 ± 14.92efg (1310.05–1457.24)	79.56 ± 9.02bcd (39.94–122.29)	29.90 ± 1.85hi (23–40)	Absent	Less distinct	Cortex
*C. carandas*	2375.92 ± 47.18 (2209.14–2606.97)	Outermost, broken, irregular cork zone. Cork layers are less lignified	262.90 ± 12.96 cd (213.11–335.14)	Wide, compactly packed cortex with more than 20 cells layered parenchymatous zone Prismatic crystals, large sclereids bands, starch grains, and broad secretory canals are present	195.56 ± 21.47a (96.44–322.38)	112.00 ± 9.51b (58.62–155.31)	Less distinct	895.32 ± 21.48bc (781.82–997.54)	Fibrillar part with numerous uniformely distributed, solitary xylem vessels	2003.36 ± 205.53bc (1277.89–2728.54)	80.1 ± 8.14bcd (34.38–114.95)	54.40 ± 3.89 fg (46–85)	Absent	Distinct	Cortex and medullary rays
*C. spinarum*	2074.49 ± 55.94 (1747.16–2286.61)	Outermost, broken, irregular, rectangular, less lignified to parenchymatous cell layers	95.61 ± 8.52 fg (90.32–142.41)	Wide compact parenchymatous zone, up to 20 cells layered Large sclereids bands, broad secretory canals, prismatic crystals, and starch grains are present	231.21 ± 31.70a (113.09–443.97)	148.27 ± 18.81a (77.37–275.02)	Less distinct phloem rays present	607.34 ± 50.79de (434.32–892.25)	Central part fibrillar and with xylem vessels Numerous uniformly distributed, solitary xylem vessels	1035.25 ± 50.35 h (802.46–1332.71)	37.89 ± 3.56d (22.88–50.73)	116.70 ± 3.20 cd (106–137)	Absent	Distinct	Cortex and medullary rays
*C. roseus*	1385.37 ± 42.57 (1217.21–1640.61)	Outer few layers of cork zone slightly lignified	113.45 ± 10.75efg (80.38–202.62)	Compact parenchymatous zone Secretory canals are compact and deformed	43.62 ± 5.90b (19.96–91.27)	23.03 ± 2.56c (14.57–41.87)	Less distinct from cortex zone	88.17 ± 4.78i (70.73–123.70)	Xylem forms a major part in the center Uniformly distributed xylem vessels present solitary and also in groups	1258.91 ± 18.13fgh (1334.01–1187.82)	30.95 ± 2.02d (21.87–42.08)	481.50 ± 7.52a (440–520)	Absent	Narrow, thin, or less distinct	Cortex
*C. dubia*	1235.64 ± 25.25 (1210.8–1324.31)	Much lignified with compactly packed cells, which generally slough off in T.S	49.84 ± 2.57 g (40.34–60.21)	Up to 15–20 cell layered parenchymatous zone with oval-shaped cells filled with starch grains Small secretory canals are present	43.07 ± 3.98b (31.24–66.96)	37.82 ± 3.72c (24.31–63.47)	Distinct phloem rays present	593.74 ± 17.23de (523.20–673.80)	Central part nearly circular, xylem with fibrillar part Scattered vessels of variable lumen size, being present singly and also in groups	488.69 ± 21.19i (378.94–591.61)	45.45 ± 7.06 cd (25.61–84.93)	108.20 ± 1.45d (100–115)	Absent	Narrow or thin	Cortex and medullary rays
*H. indicus*	1853.75 ± 26.73 (1692.34–1963.24)	Much lignified zone forming a clear dark outer portion	117.98 ± 5.09efg (97.14–144.03)	Parenchymatous thick zone up to 20 layered, formed of compactly packed cells Calcium oxalate crystals are present in some cells Small secretory canals are present	44.32 ± 5.42b (24.34–80.76)	26.97 ± 2.75c (15.10–43.44)	Distinct compact zone	692.32 ± 25.26d (523.07–801.19)	The major central part formed of xylem fibers Scattered xylem vessels of variable lumen size, present solitary or in groups	1021.98 ± 18.85 h (947.30–1106.82)	57.43 ± 8.62 cd (93.84–16.40)	74.30 ± 2.46e (62–91)	Absent	Narrow or thin	Cortex and medullary rays
*H. pubescens*	1983.01 ± 29.82 (1887.49–2122.16)	Outer thin and lignified layer	77.25 ± 5.74 fg (56.30–106.14)	Parenchymatous cell zone, up to 15 layered Small secretory canals are present	43.77 ± 3.72b (27.80–56.96)	32.57 ± 2.41c (18.82–42.61)	Distinct compact zone	570.17 ± 11.58ef (531.92–648.09)	Major fibrillar zone Several scattered vessels, uniformly distributed being present in groups and rows	1716.92 ± 33.29 cd (1449.00–1796.27)	74.43 ± 7.73bcd (39.66–114.83)	111.40 ± 2.87 cd (100–132)	Very small pith present	Distinct	Cortex and medullary rays
*I. frutescens*	2704.84 ± 25.91 (2558.15–2814.93)	Thin, compact, and lignified outer portion	51.59 ± 3.68 g (38.31–68.31)	Up to 25–30 cells layered parenchymatous zone, filled with starch grains, with brown coloured cell contents Small secretory canals are present	41.93 ± 1.62b (32.33–48.21)	25.83 ± 1.62c (16.53–34.08)	Distinct phloem rays present	864.42 ± 22.13c (743.24–958.81)	The major part formed of xylem vessels with the wide lumen and also fibrillar zone Most xylem vessels with a wide lumen present solitary and in groups	1488.50 ± 14.58def (1432.75–1574.61)	114.79 ± 20.34ab (33.71–210.02)	45.90 ± 1.58 g (40–55)	Present	Narrow or thin	Cortex and medullary rays
*M. tenacissima*	2503.47 ± 36.20 (2350.96–2669.68)	Present in broken patches with cork layers less distinguishable. Few outer cork layers with sclereids and lignified patches were observed	77.27 ± 4.71 fg (60.31–103.13)	Up to 30–40 cell layered parenchymatous zone, cells with starch grains Large sclereids bands are present Compact, small, and deformed secretory canals are present	53.28 ± 4.42b (41.51–88.53)	42.24 ± 1.51c (34.19–49.22)	Distinct phloem rays present	1420.88 ± 20.80a (1315.21–1513.29)	Xylem zone with angular outline present in the center, consisted of xylem fibrillar zone and xylem vessels Uniformly scattered xylem vessels of variable lumen size, present solitary and in groups	1227.09 ± 85.39fgh (930.92–1665.34)	90.99 ± 13.47abc (46.91–196.05)	56.10 ± 1.92 fg (49–71)	Absent	Distinct	Cortex and medullary rays
*N. oleander*	3983.43 ± 38.40 (3794.68–4163.79)	Much lignified broken flaky layers	170.84 ± 13.87def (111.12–234.03)	Up to 20 cells layered parenchymatous zone formed of compact cells Small secretory canals are present	49.56 ± 4.76b (26.85–82.15)	28.18 ± 3.58c (16.80–54.42)	Distinct compact zone present	420.29 ± 15.83 g (348.69–487.72)	Major fibrillar zone Vessels scattered, with small lumen size and linear arrangement	3393.11 ± 57.89a (3131.22–3590.64)	54.92 ± 5.79 cd (29.26–81.55)	43.80 ± 1.30gh (37–49)	Very small pith present	Narrow or thin	Cortex
*R. serpentina*	1970.86 ± 28.35 (1839.27–2096.41)	Thick zone with rectangular parenchymatous cells	385.56 ± 30.06b (191.06–485.11)	Up to 20 cells layered cortex zone with compactly packed parenchymatous cells filled with starch grains Small secretory canals are present	57.55 ± 2.54b (46.25–67.32)	28.41 ± 1.98c (19.95–42.97)	Thin or less distinct phloem zone	475.87 ± 12.21 fg (415.2–523.44)	Xylem consisted of numerous uniformly distributed, small-sized xylem vessels present in the xylem fiber zone Xylem vessels present solitary, in groups, and linear arrangement	1062.98 ± 17.34gh (962.29–1126.20)	32.02 ± 1.59d (25.47–41.06)	152.40 ± 1.95b (140–160)	Absent	Distinct	Cortex and medullary rays
*T. divaricata*	2244.62 ± 28.09 (2149.81–2389.04)	Thick zone with compact parenchymatous cells and outer few cells lignified	209.35 ± 8.83de (156.02–241.22)	Compact parenchymatous cortex zone, up to 20 cell layered Small secretory canals are present	54.05 ± 7.52b (27.28–104.73)	24.24 ± 2.79c (11.20–41.54)	A crushed or compact, thin zone	438.60 ± 24.82 g (312.49–551.08)	Major fibrillar zone Uniformly distributed xylem vessels numerous being present solitary, in groups and a linear arrangement	1630.97 ± 29.04de (1465.88–1711.92)	43.28 ± 6.88 cd (20.28–84.19)	123.50 ± 1.57c (115–131)	Absent	Distinct	Medullary rays

*S.E.*  Standard Error, *n*  Number of samples, *C*  Cortex, *P*  Phloem; *C + P* Cortex plus Phloem, *T.S.* Transverse Section

One-way ANOVA’s were carried out separately for each quantitative character to figure out the differences among different species. The same letters after values in a column denote a lack of statistically significant differences, according to Tukey’s post hoc test (p < 0.05)

## Chemical analysis

### TLC chromatogram profile

**Fig. 6:**
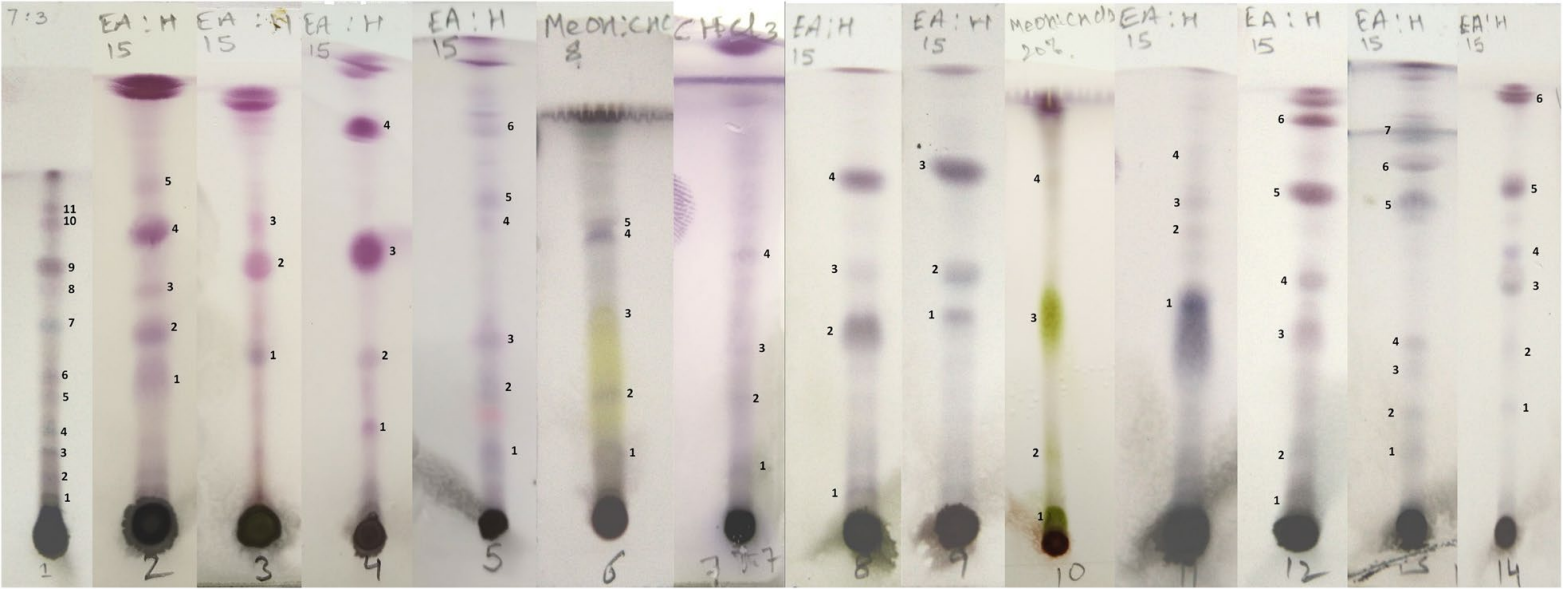
TLC profiles of root crude extracts of 14 species of Apocynaceae (TLC profiles numbered from 1 to 14 represent species *A. curassavica* to *T. divaricata* respectively)

TLC was employed for the preliminary phytochemical investigation of the crude extracts of root samples under study. For development of TLC profile of root crude extracts of 14 species, literature review was done and accordingly varied combinations of solvents were used for chromatographic separation of chemical constituents of crude extract. Out of various combinations performed, the most suitable combinations of solvents with most desirable results are shown in Table 6. The TLC of root samples of studied 14 species were observed with prominent bands with different retention factor (R*f*) values (Table 6). The migration profiles of constituents of the root samples are shown in Fig. 6.

**Table 6:** Table showing solvent system, migration profiles of solvent and compounds along with R_*f*_ values for the crude exracts of root samples of 14 species of family Apocynaceae

Species name	Sp 1 (*A. curassavica*)		Sp 2 (*C. gigantea*)		Sp 3 (*C. procera*)		Sp 4 (*C. carandas*)		Sp 5 (*C. spinarum*)		Sp 6 (*C. roseus*)		Sp 7 (*C. dubia*)	
Solvent system (ratio)	Ethyl acetate:Hexane (3:7)		Ethyl acetate:Hexane (1.5:8.5)		Ethyl acetate:Hexane (1.5:8.5)		Ethyl acetate:Hexane (1.5:8.5)		Ethyl acetate:Hexane (1.5:8.5)		MeOH:CHCl3 (0.8:9.2)		CHCl3	
Total run (in cm)	6.7		7.5		7.3		8.0		8.3		6.8		7.5	
S.no	Band distance (in cm)	R_*f*_ value	Band distance (in cm)	R_*f*_ value	Band distance (in cm)	R_*f*_ value	Band distance (in cm)	R_*f*_ value	Band distance (in cm)	R_*f*_ value	Band distance (in cm)	R_*f*_ value	Band distance (in cm)	R_*f*_ value
1	0.8	0.12	2.6	0.35	2.9	0.40	1.9	0.24	1.5	0.18	1.3	0.19	1.0	0.13
2	1.2	0.18	3.3	0.44	4.4	0.60	3.0	0.38	2.5	0.30	2.2	0.32	2.3	0.31
3	1.6	0.24	4.0	0.53	5.2	0.71	4.9	0.61	3.2	0.39	3.5	0.51	3.1	0.41
4	2.0	0.30	5.0	0.67			6.7	0.84	5.2	0.63	4.8	0.71	4.7	0.63
5	2.7	0.40	5.7	0.76					5.6	0.67	5.0	0.74		
6	3.0	0.45							6.8	0.82				
7	3.9	0.58												
8	4.6	0.69												
9	5.0	0.75												
10	5.7	0.85												
11	6.0	0.90												

Species name	Sp 8 (*H. indicus*)		Sp 9 (*H. pubescens*)		Sp 10 (*I. frutescens*)		Sp 11 (*M. tenacissima*)		Sp 12 (*N. oleander*)		Sp 13 (*R. serpentina*)		Sp 14 (*T. divaricata*)	
Solvent system (ratio)	Ethyl acetate:Hexane (1.5:8.5)		Ethyl acetate:Hexane (1.5:8.5)		MeOH:CHCl3 (0.8:9.2)		Ethyl acetate:Hexane (1.5:8.5)		Ethyl acetate:Hexane (1.5:8.5)		Ethyl acetate:Hexane (1.5:8.5)		Ethyl acetate:Hexane (1.5:8.5)	
Total run (in cm)	7.6		7.7		7.3		7.6		7.4		7.7		7.5	
S.no	Band distance (in cm)	R_*f*_ value	Band distance (in cm)	R_*f*_ value	Band distance (in cm)	R_*f*_ value	Band distance (in cm)	R_*f*_ value	Band distance (in cm)	R_*f*_ value	Band distance (in cm)	R_*f*_ value	band distance (in cm)	R_*f*_ value
1	0.8	0.11	3.7	0.48	0.5	0.07	3.9	0.51	0.7	0.09	1.3	0.17	2.2	0.29
2	3.5	0.46	4.4	0.57	1.5	0.21	5.1	0.67	1.4	0.19	2.0	0.26	3.1	0.41
3	4.4	0.58	6.1	0.79	3.7	0.51	5.6	0.74	3.3	0.45	2.7	0.35	4.1	0.55
4	5.8	0.76			5.9	0.81	6.4	0.84	4.2	0.57	3.2	0.42	4.7	0.63
5									5.7	0.77	5.5	0.71	5.8	0.77
6									6.8	0.92	6.1	0.79	7.2	0.96
7											6.7	0.87

### LC–MS profile

**Fig. 7:**
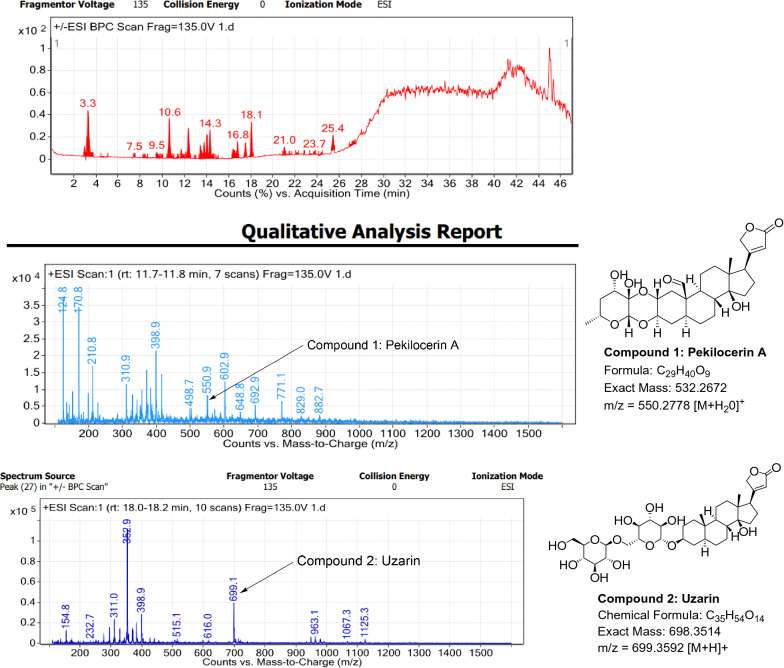
LC–MS chromatogram of root extract of *Asclepias curassavica* showing marker compounds

**Fig. 8:**
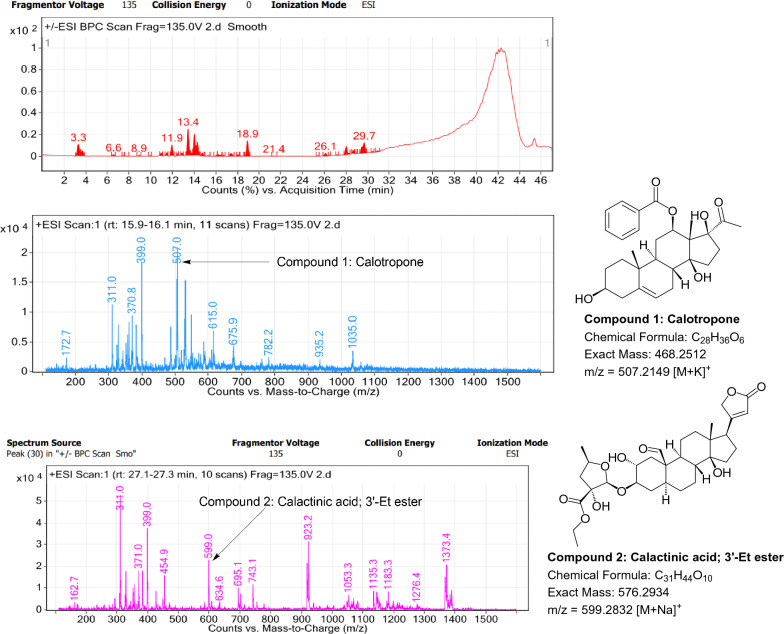
LC–MS chromatogram of root extract of *Calotropis gigantea* showing marker compounds

**Fig. 9:**
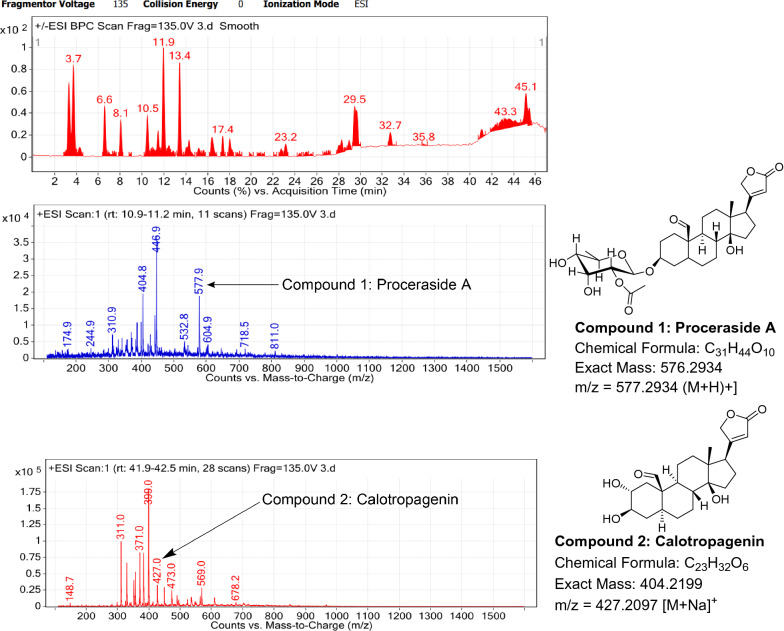
LC–MS chromatogram of root extract of *Calotropis procera* showing marker compounds

**Fig. 10:**
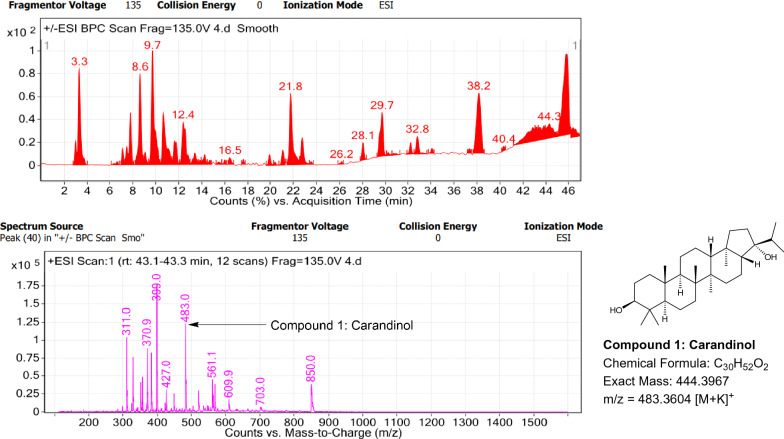
LC–MS chromatogram of root extract of *Carissa carandas* showing marker compounds

**Fig. 11:**
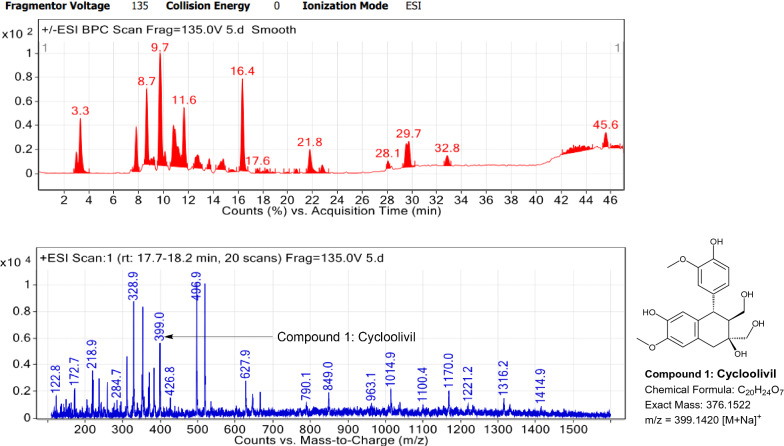
LC–MS chromatogram of root extract of *Carissa spinarum* showing marker compounds

**Fig. 12:**
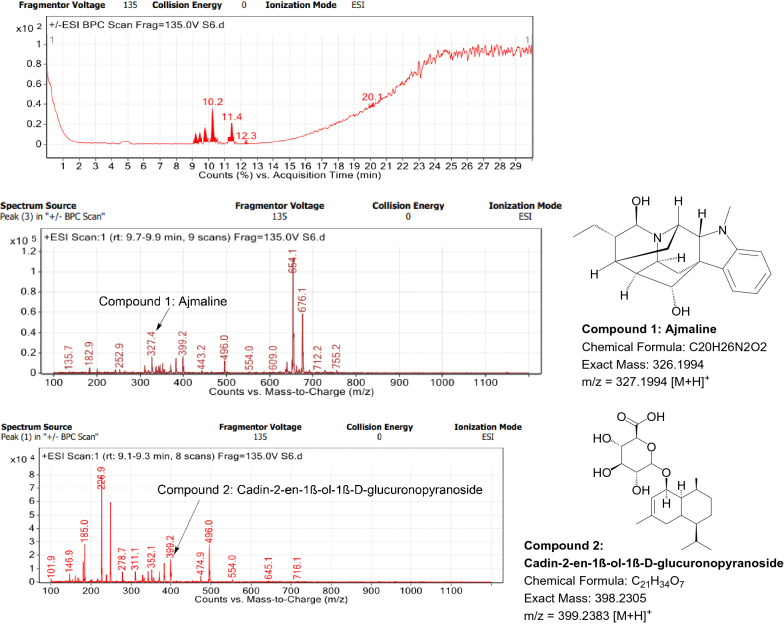
LC–MS chromatogram of root extract of *Catharanthus roseus* showing marker compounds

**Fig. 13:**
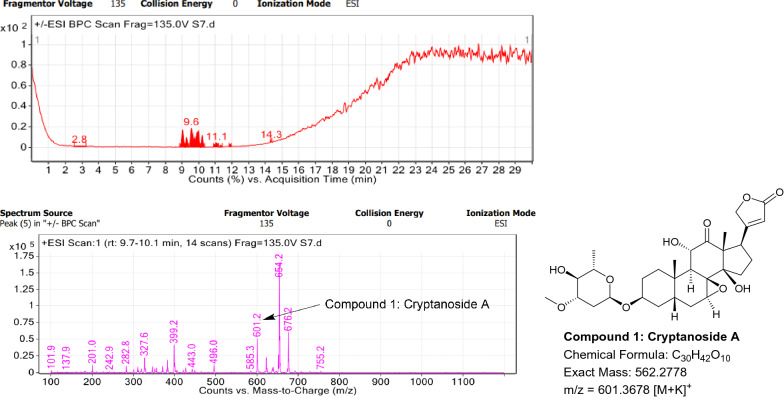
LC–MS chromatogram of root extract of *Cryptolepis dubia* showing marker compounds

**Fig. 14:**
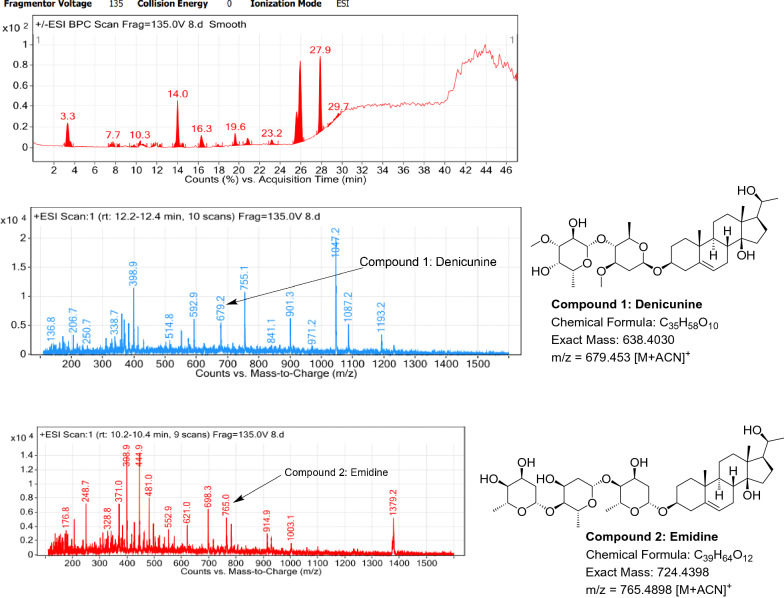
LC–MS chromatogram of root extract of *Hemidesmus indicus* showing marker compounds

**Fig. 15:**
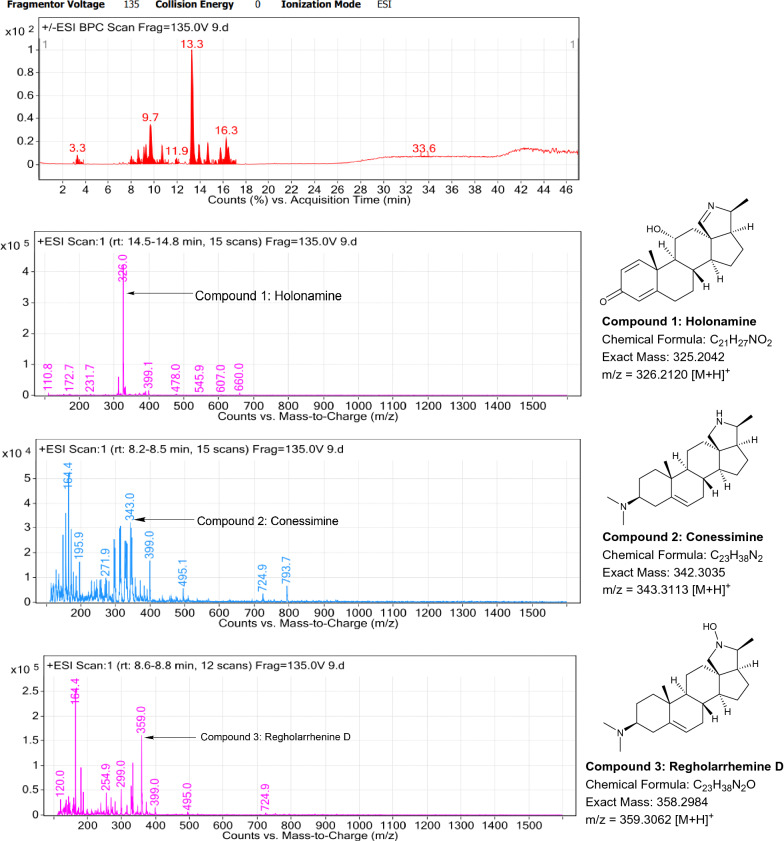
LC–MS chromatogram of root extract of *Holarrhena pubescens* showing marker compounds

**Fig. 16:**
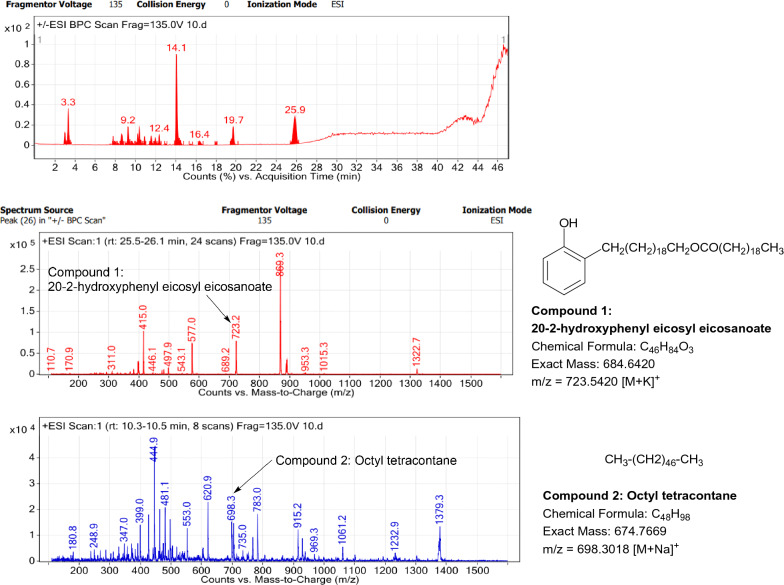
LC–MS chromatogram of root extract of *Ichnocarpus frutescens* showing marker compounds

**Fig. 17:**
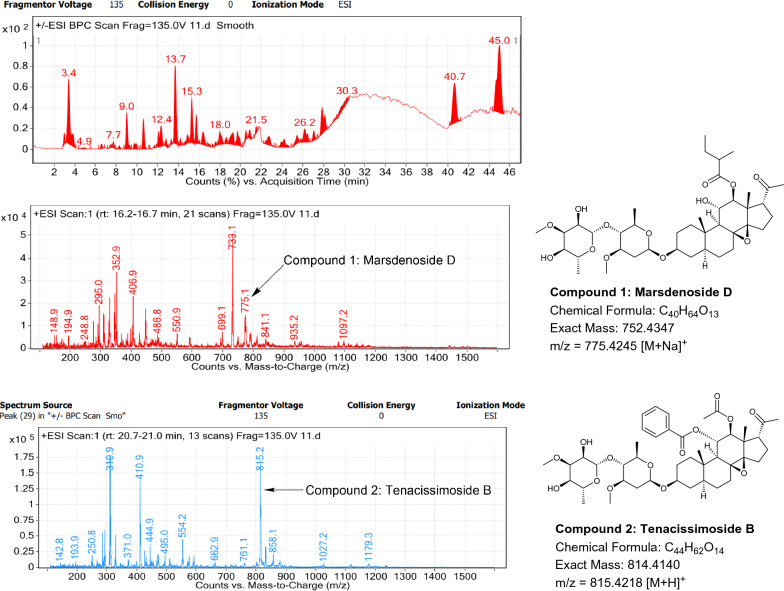
LC–MS chromatogram of root extract of *Marsdenia tenacissima* showing marker compounds

**Fig. 18:**
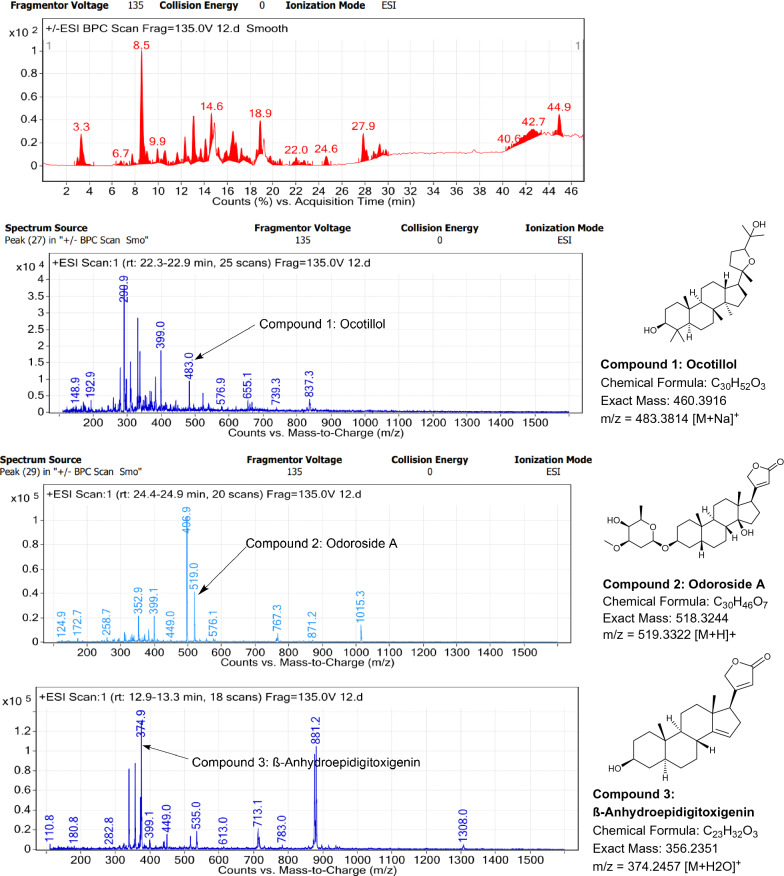
LC–MS chromatogram of root extract of *Nerium oleander* showing marker compounds

**Fig. 19:**
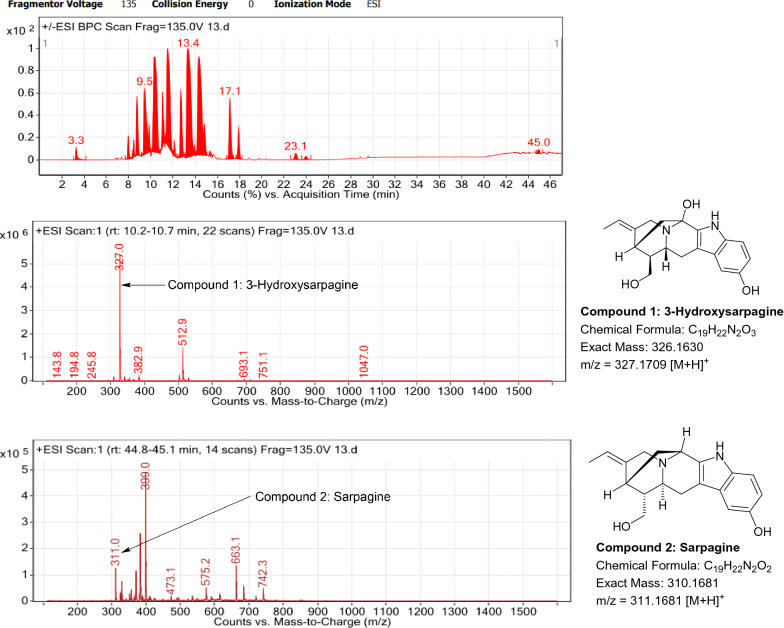
LC–MS chromatogram of root extract of *Rauvolfia serpentina* showing marker compounds

**Fig. 20:**
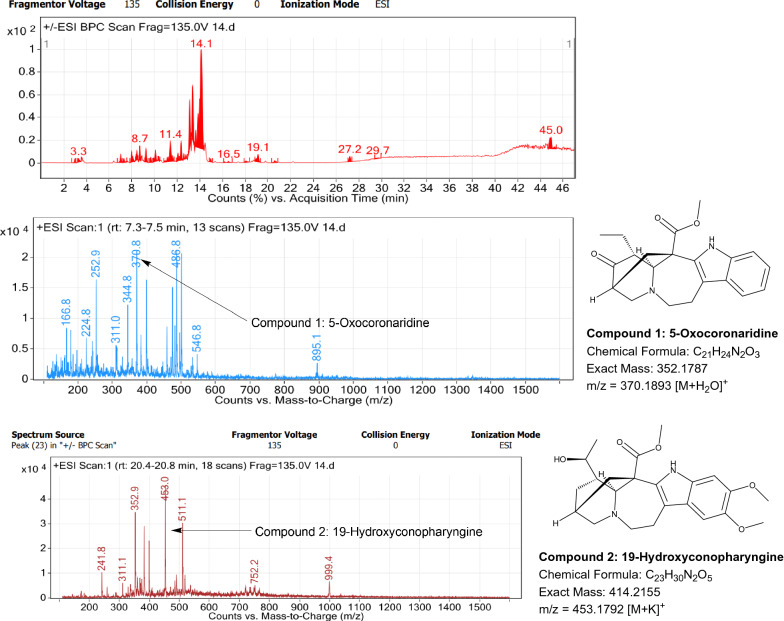
LC–MS chromatogram of root extract of *Tabernaemontana divaricata* showing marker compounds

The dataset generated by Liquid Chromatography mass spectrometry measurement of raw plant materials can be used for authentication of plant species. In present study, LC–MS analysis compared the phytochemical contents of the methanol extracts of root samples. Some plant metabolites were identified for each of the 14 species in a single analytical run (Table 7), which helped in species identification with high accuracy. Although it was difficult to identify each peak in the LC–MS chromatogram, some major constituents were identified for studied species. The characteristic compounds from crude extracts of the given plants have been identified by the LC–MS technique. In LC–MS studies of root extract, the major compounds were identified based on its mass data and UV pattern. The chemical constituents for 14 different species at specific retention time are given in Figs. 7, 8, 9, 10, 11, 12, 13, 14, 15, 16, 17, 18, 19, 20.

**Table 7:** Table showing the list of compounds identified by LC–MS studies from the root crude drug samples of 14 species of family Apocynaceae

S. n.o	Species name	Compounds identified by LC–MS (References)
1.	*Asclepias curassavica* L.	1: Pekilocerin A (Kiuchi et al. 1998) 2: Uzarin (Hanna et al. 1999)
2.	*Calotropis gigantea* (L.) Dryand	1: Calotropone (Wang et al. 2008) 2: Calactinic acid; 3'-Et ester (Roy et al. 2005; Seeka and Sutthivaiyakit 2010)
3.	*Calotropis procera* (Aiton) Dryand	1: Proceraside A (Ibrahim et al. 2014) 2: Calotropagenin (Seeka and Sutthivaiyakit 2010)
4.	*Carissa carandas* L.	1: Carandinol (Begum et al. 2013)
5.	*Carissa spinarum* L.	1: Cycloolivil (wahab Sab et al. 2015)
6.	*Catharanthus roseus* (L.) G. Don	1: Ajmaline (Itoh et al. 2005) 2: Cadin-2-en-1β-ol-1β-D-glucuronopyranoside (Chung et al. 2007)
7.	*Cryptolepis dubia* (Burm.f.) M.R.Almeida	1: Cryptanoside A (Purushothaman et al. 1988)
8.	*Hemidesmus indicus* (L.) R. Br. ex Schult	1: Denicunine (Sigler et al. 2000) 2: Emidine (Chandra et al. 1994)
9.	*Holarrhena pubescens* Wall. ex G.Don	1: Holonamine (Nnadi et al. 2017) 2: Conessimine (Nnadi et al. 2017) 3: Regholarrhemine D (Bhutani et al. 1990)
10.	*Ichnocarpus frutescens* (L.) W.T.Aiton	1: Octyl tetracontane (Aggarwal et al. 2010) 2: 20-(2-Hydroxyphenyl) eicosyl eicosanoate (Aggarwal et al. 2010)
11.	*Marsdenia tenacissima* (Roxb.) Moon	1: Marsdenoside D (Deng et al. 2005) 2: Tenacissimoside B (Jiang and Luo 1996)
12.	*Nerium oleander* L.	1: Ocotillol (Tanaka et al. 1993) 2: Odoroside A (Isobe et al. 1986; Abe et al. 1996) 3: β-Anhydroepidigitoxigenin (Huq et al. 1999)
13.	*Rauvolfia serpentina* (L.) Benth ex Kurz	1: 3-Hydroxysarpagine (Rukachaisirikul et al. 2017) 2: Sarpagine (Rukachaisirikul et al. 2017)
14.	*Tabernaemontana divaricata* (L.) R.Br. ex Roem. & Schult.	1: 5-Oxocoronaridine (Liu et al. 2016) 2: 19-Hydroxyconopharyngine (Zocoler et al. 2005)

## Discussion

Morphological features (shape, size, color, surface feature, texture, fracture, and appearance) and anatomical features are considered of diagnostic value in the identification and distinction of herbal drug samples in several plant groups (Fritz and Saukel 2011; Manohan et al. 2013; Ginko et al. 2016; Park et al. 2019). Surface characters may not be used for species authentication; however, the combination of some surface and cut root surface characters can be used in the preliminary distinction of samples. According to Park et al. (2019), the only morphological character-based distinction of root drug samples is challenging and also requires anatomical characterisation. In the anatomical study, principal dominating tissue in cross-section and other tissue zone was considered a suitable character for distinguishing herbal drug samples (Fritz and Saukel 2011; Hassan et al. 2015; Ginko et al. 2016). In the present anatomical study, the relative thickness, number & arrangement of cell layers of outer bark (cork region), inner bark (cortex and phloem), and woody zone (xylem) to the total radius of the studied T.S. were observed varying for studied species (Fig. 3).

Some bark anatomical features such as the structure of cork, number, and thickness of cork layers, the occurrence of sclereids, type, structure, and arrangement of secretory ducts, presence of crystals were also known of taxonomic value in species characterization (Fritz and Saukel 2011; Ginko et al. 2016; Park et al. 2019). In the present study, the cork zone was variable in colour and lignifications of cell walls, cortex zone varied in the cell composition, cell contents, mean lumen size of secretory canals, and occurrence of sclereids. The cortex of some species was observed with characteristic anatomical features. For example, distinct large sclereids bands and secretory canals in two *Carissa* spp., broad sclereid patches in *M. tenacissima*, and reddish-brown colored patches in *I. frutescens* (Figs. 1, 2). A comparative morphometric study in the present study revealed variation in mean lumen diameter of secretory canals in both *Carissa* spp. (comparatively broader in *C. spinarum*). In the present study, anatomical characters of root bark of *M. tenacissima* corresponded with anatomical structure observed by Tripathi et al. (2014). Anatomical characters of the *Carissa* genus corresponded with anatomical characters in some previous studies (Salunke and Ghate 2013; Khalil et al. 2015; Allam et al. 2016).

Some vascular anatomical characters such types, arrangement, and grouping of xylem vessels, vessel outline, the dimension of largest vessels, frequency of vessels per square area; the appearance of medullary rays in secondary xylem, the thickness of medullary rays, and laticifers in rays, etc. were reported as significant characters in discrimination of different root samples (Lens et al. 2008; Fritz and Saukel 2011; Ginko et al. 2016; Park et al. 2019). In the present study, xylem anatomical characters such as mean lumen diameter of xylem vessels, number of vessels per square area, xylem vessel arrangement, and distribution were observed as the variable for various species under study. The mean lumen diameter of xylem vessels in the studied species ranged from 30.95 µm (*C. roseus*) to 134.54 µm (*C. gigantea*). The mean number of vessels per square area ranged from 22.8 (*C. gigantea*) to 481.5 (*C. roseus*). Quantitative xylem anatomical characters for other species are shown in Table 5.

Powder microscopy helps identify broken or powdered plant samples (Sereena and Sreeja 2014). Several microscopic features, including starch grains and crystals types, are considered helpful in identifying some herbal material (Cortella and Pochettino 1994; Lens et al. 2008; Ginko et al. 2016; Ya’ni et al. 2018). The organoleptic examination of root powder samples showed variation in color, odor, texture, and taste in the present study. Organoleptic and microscopic characters of powder are provided in Table 4 and Additional file 1: Fig. S1–S2. Species belonging to the same genus were observed with comparatively more similarities in powder characters. Organoleptic and microscopic powder characters of two *Carissa* spp. and *Calotropis* spp. were nearly identical. In powder study, variation was observed in quantitative and quantitative microscopic features such as the shape and size of starch grains, the grouping of starch grains; the type of crystals; the size of prismatic crystals, the abundance of coloured fragments. An iodine test in T.S. of the root also showed variation in abundance and distribution of starch grains in a different zone of T.S. of the root (Additional file 1: Fig. S1–S2).

The powder sample of two *Calotropis* spp. showed variation only in taste, mean size of starch grains and colorful crystals, and abundance of starch grains in medullary rays. Some other genera (such as *C. dubia*, *H. indicus,* and *I. frutescens*, all three with the common name ‘Sariva’) were similar in some organoleptic and microscopic powder characters. However, the color of powder samples and the shape of starch grains also varied (circular in *C. dubia*, oval to slightly elongated in *H. indicus*, and oval to circular in *I. frutescens*).

Among the studied species, some previous studies had been done on some species. Out of the 14 species studied, macro and microscopic identification studies for 11 species were conducted earlier by various researchers. Root anatomical studies were performed earlier in *A. curassavica* (Hassan et al. 1952; Kalidass et al. 2009a; Ramesh et al. 2014); *C. gigantea* (Shirsat et al. 2011); *C. procera* (API 2001; Hassan et al. 2015); *C. carandas* (API 2001; Mishra et al. 2013; Salunke and Ghate 2013); *C. spinarum* (Salunke and Ghate 2013); *C. dubia* (API 2001; Jeewandara et al. 2017); *H. indicus* (API 2001; Chitra and Thoppil 2002; Shanthi et al. 2010; Rajan et al. 2011; Sariga and Shajahan 2017; Jeewandara et al. 2017); *I. frutescens* (Kalidass et al. 2009b; Jeewandara et al. 2017); *M. tenacissima* (API 2001; Tripathi et al. 2014; Kolhe et al. 2014); *N. oleander* (API 2001) and *R. serpentina* (API 2001; Panda et al. 2012; Rungsung et al. 2014). However, root anatomical studies on *C. roseus*, *H. pubescens,* and *T. divaricata* have been performed for the first time in the present study. Of the several previous identification studies performed on root samples, the majority of research focused on a qualitative description of macroscopic, anatomical, and powder characters with sketch diagrams or some with cross-section photographs; however, quantitative characterization was sparse in most studies (Salunke and Ghate 2013; Jeewandara et al. 2017; Hassan et al. 2015). The present study provided a detailed comparative macroscopic and microscopic study of root samples, including a description of qualitative and quantitative characters with corresponding images.

Statistical analysis revealed some significant characters with taxonomic importance in species distinction. Microscopic characters and character states have been used in phenetic analysis and systematic study of many plant species (Ginko et al. 2016; Ya’ni et al. 2018). In the present study, the grouping of most species was observed nearly congruent to some previously published classifications (Lens et al. 2008; Nazar et al. 2013; Endress et al. 2014). The closely grouped species in dendrogram can be expected with greater chances of adulteration. In the group of three species (*C. dubia*, *H. indicus*, and *I. frutescens*), the official part of the drug ‘Sariva’ (*H. indicus*) is reported to be adulterated by the root samples of other two genera with the similar common name (Jeewandara et al. 2017). Other species belonging to the same genus (*Calotropis* spp. and *Carissa* spp.) were observed in a close clade. Such closely grouped species can be distinguished based on some unique combination of botanical characters identified in the present study.

In the present study, some surface and anatomical characters such as the appearance of bark and the presence or absence of pith, etc. observed as less stable and should be carefully considered for the identification of herbal samples. For example, root bark was observed sloughed off in dried root samples of *C. dubia*. Similarly, Jeewandara et al. (2017) observed pith in older roots of *C. dubia*; however, in the present study, pith was not observed. The physical integrity of raw herbal samples is considered essential as identifying herbal drugs only from powdered samples can be challenging (Ginko et al. 2016). In addition, a single botanical character may not be considered unique in describing a species. For plant species with similar botanical features, a combination of diagnostic microscopic characters is essential for species identification and distinction of herbal samples (Lens et al. 2008, 2009; Ginko et al. 2016). Detailed taxonomic information provided in the present study can be helpful in taxonomic identification and distinction of genuine raw herbal drugs from contaminants to be used for herbal drug preparations. Chemical profiling of herbal samples in addition to botanical characterization is helpful and is more authentic in the identification of raw herbal drugs.

In the present study, the TLC fingerprinting profile was done for methanolic extracts of root samples of selected 14 species of family Apocynaceae. The R_*f*_ values acquired from TLC chromatograms provided essential information regarding their polarity of phytochemicals as well as important clues in the separation process. The usage of multiple solvent systems for TLC investigations could be essential for selecting the suitable solvent system since different R_*f*_ values of the molecule reflect a notion about their polarity. This knowledge will aid in the selection of a suitable solvent system for subsequent compound separation from these plant extracts. However, the TLC results were not sufficient to determine their profile and the chemical complexity of the crude extracts. Thus to identify phytoconstituents in root extracts, Liquid Chromatography-Mass Spectrometry (LC–MS) studies were also carried out in present work. LC–MS analysis is now a routine technique employed to identify phytoconstituents present in a wide range of botanical samples (Zhao et al. 2005; Lai et al. 2015; Park et al. 2019). Park et al. (2019) performed LC–MS profiling alongwith anatomical studies to develop identification standards of roots of *Adenophora* sp. In the present study, the chemical compounds identified were major metabolites present in 14 species and were comparable with literature reports (Table 7). In addition, the NMR data is also obtained for the identified compounds which are comparable to published reports (data provided in supplementary file as ‘Additional file 1’). These compounds provide supportive data can be used as the chemical markers for the identification of raw herbal drugs in addition to botanical data.

While modern testing techniques for evaluating plant drugs are available in today's scientific age, microscopic analysis remains one of the most basic and cost-effective methods for correctly identifying source materials (Kumar et al. 2011). Anatomical studies are helpful in the distinction of herbal samples with similar morphological characters (Traiperm et al. 2017). The combined approach involving botanical and chemical identifcaiton adopted in the present study ensures more authenticity in sample identification irrespective the physical form of herbal sample. The identification standards thus help overcome the adulteration and misidentification problems.

## Conclusions

Detailed comparative botanical characterisation (qualitative and quantitative features) of root drug samples was found helpful in identifying and distinguishing similar-looking adulterant samples. Statistical analysis of botanical characters helped in identification of some of taxonomically significant characters in distinction of root samples. Among various characters, the clustering of xylem vessels was observed as the most significant character in Apocynaceae species’ distinction from PCA values. The unique chromatographic fingerprint profiles and major chemical constituents identified for studied species further aid in distinction of root samples of closely related species. The combined study including botanical and chemical characterization in the present study provide a reference database for future identification of raw root samples. The studies performed in present study will help the herbal industry in quality control of raw herbal drugs and the botanical characters further help as a reference guide for future taxonomic studies of herbal drugs.

## Supplementary Information

**Additional file 1**. NMR spectroscopic data of all the identified marker compounds from the crude root extracts of fourteen species of family Apocynaceae. **Fig. S1**. Powder characteristics of the RDS of seven studied roots of the family Apocynaceae used in ISM (*A. curassavica* to *C. dubia*). **Fig. S2**. Powder characteristics of the RDS of the studied seven roots of the family Apocynaceae used in ISM (*H. indicus* to *T. divaricata*). **Table S1**. Data matrix showing codes for the studied RDS of family Apocynaceae used in ISM.

## Abbreviations

RDS: Root drug samples
PCA: Principal component analysis
ISM: Indian systems of medicine
MT: Metric tonnes
NA: Not available
S.E.: Standard error
Dil: Digitalose
Ole: Oleandrose
