# Supplementary material for: Comparative morpho-anatomical standardization and chemical profiling of root drugs for distinction of fourteen species of family Apocynaceae
Source: Bot Stud. 2022 Apr 25;63:12. doi: 10.1186/s40529-022-00342-z (PMC9038984; doi:10.1186/s40529-022-00342-z)
Supplement: Supplementary file 1 — Additional file 1. NMR spectroscopic data of all the identified marker compounds from the crude root extracts of fourteen species of family Apocynaceae. Fig. S1. Powder characteristics of the RDS of seven studied roots of the family Apocynaceae used in ISM (A. curassavica to C. dubia). Fig. S2. Powder characteristics of the RDS of the studied seven roots of the family Apocynaceae used in ISM (H. indicus to T. divaricata). Table S1. Data matrix showing codes for the studied RDS of family Apocynaceae used in ISM. [file 40529_2022_342_MOESM1_ESM.docx]

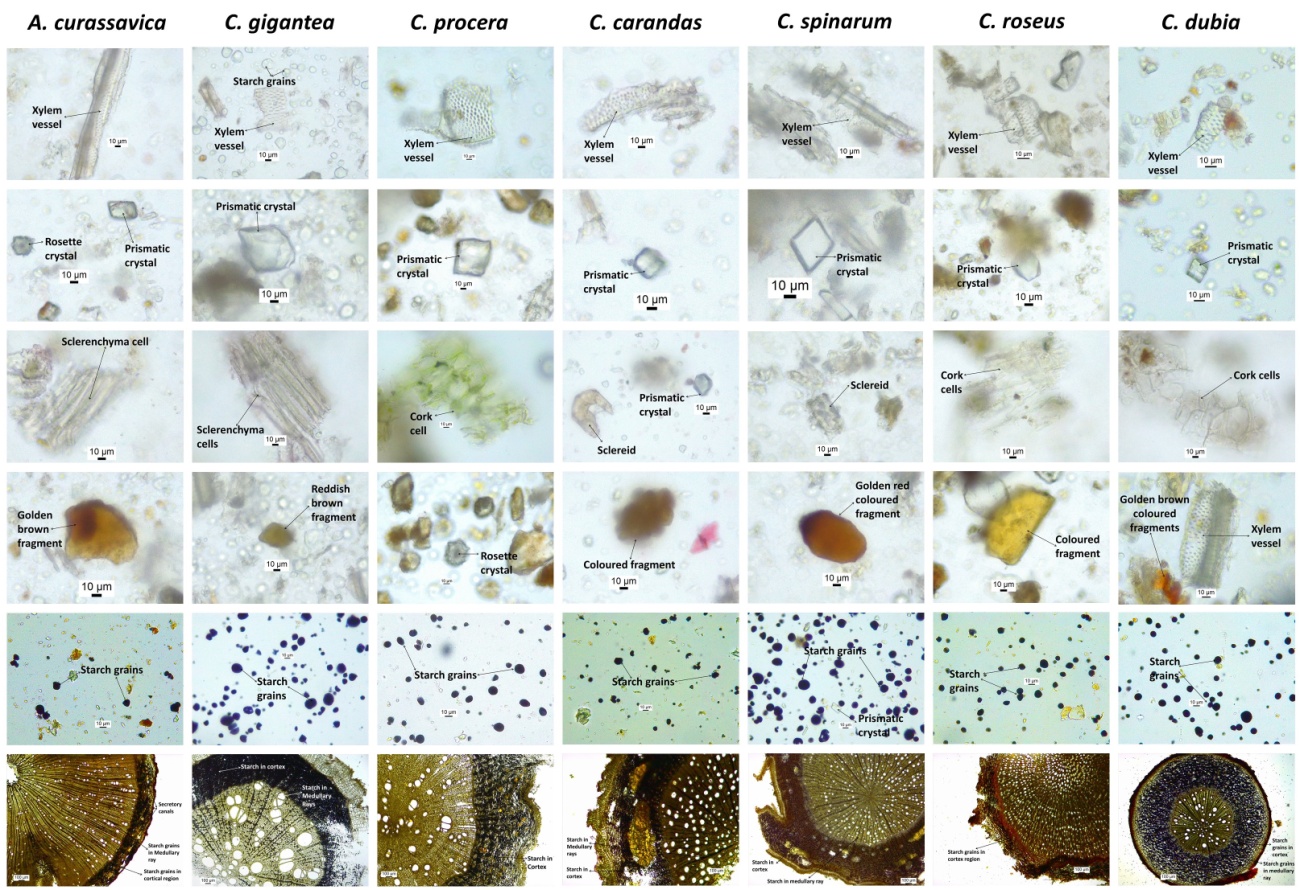


**Fig. S1** Powder characteristics of the RDS of seven studied roots of the family Apocynaceae used in ISM (*A. curassavica* until *C. dubia*).


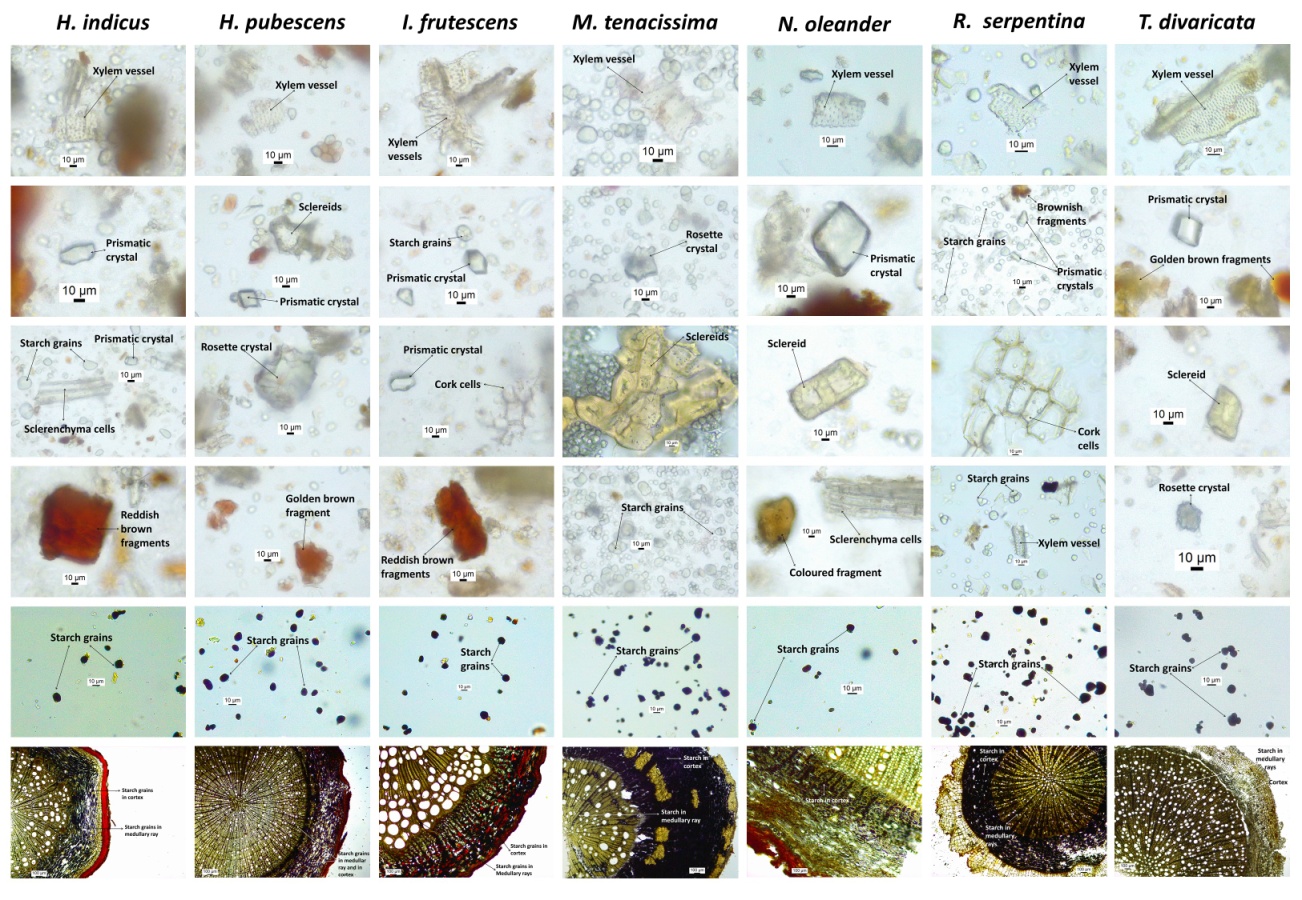


**Fig. S2** Powder characteristics of the RDS of the studied seven roots of the family Apocynaceae used in ISM (*H. indicus* until *T. divaricata*).

**Table S1** Data matrix showing codes for the studied RDS of family Apocynaceae used in ISM.

| **Species** | **Character No and Code (same as in Table 1)** | | | | | | | | | | | | | | | | | | | | | | | | | | | | | | | | | |
| --- | --- | --- | --- | --- | --- | --- | --- | --- | --- | --- | --- | --- | --- | --- | --- | --- | --- | --- | --- | --- | --- | --- | --- | --- | --- | --- | --- | --- | --- | --- | --- | --- | --- | --- |
|  | **1** | **2** | **3** | **4** | **5** | **6** | **7** | **8** | **9** | **10** | **11** | **12** | **13** | **14** | **15** | **16** | **17** | **18** | **19** | **20** | **21** | **22** | **23** | **24** | **25** | **26** | **27** | **28** | **29** | **30** | **31** | **32** | **33** |  |
| ***A. curassavica*** | 1 | 2 | 2 | 2 | 2 | 2 | 1 | 2 | 3 | 2 | 2 | 2 | 5 | 2 | 1 | 1 | 1 | 1 | 2 | 1 | 6 | 3 | 2 | 4 | 2 | 1 | 2 | 4 | 2 | 3 | 1 | 1 | 1 |  |
| ***C. gigantea*** | 2 | 1 | 1 | 1 | 1 | 1 | 2 | 2 | 3 | 2 | 2 | 2 | 2 | 2 | 2 | 2 | 2 | 2 | 3 | 2 | 5 | 1 | 1 | 2 | 2 | 2 | 4 | 5 | 2 | 1 | 1 | 1 | 2 |  |
| ***C. procera*** | 2 | 1 | 1 | 1 | 2 | 1 | 2 | 2 | 2 | 2 | 2 | 2 | 2 | 2 | 2 | 1 | 2 | 2 | 3 | 3 | 5 | 1 | 2 | 4 | 2 | 2 | 2 | 5 | 2 | 4 | 2 | 1 | 2 |  |
| ***C. carandas*** | 2 | 1 | 1 | 2 | 2 | 1 | 1 | 2 | 1 | 1 | 1 | 1 | 3 | 2 | 1 | 1 | 1 | 3 | 2 | 2 | 1 | 2 | 1 | 3 | 2 | 2 | 4 | 5 | 2 | 1 | 2 | 2 | 1 |  |
| ***C. spinarum*** | 2 | 1 | 1 | 1 | 1 | 1 | 2 | 2 | 1 | 2 | 1 | 1 | 3 | 2 | 1 | 1 | 1 | 3 | 2 | 3 | 1 | 1 | 1 | 3 | 2 | 2 | 4 | 5 | 2 | 1 | 1 | 2 | 1 |  |
| ***C. roseus*** | 1 | 2 | 2 | 2 | 2 | 2 | 1 | 2 | 2 | 2 | 1 | 4 | 3 | 1 | 2 | 2 | 2 | 1 | 2 | 1 | 5 | 3 | 1 | 2 | 2 | 2 | 2 | 5 | 1 | 1 | 2 | 1 | 1 |  |
| ***C. dubia*** | 2 | 1 | 2 | 2 | 2 | 1 | 1 | 1 | 3 | 1 | 2 | 2 | 2 | 2 | 2 | 1 | 2 | 3 | 1 | 3 | 5 | 1 | 1 | 2 | 2 | 2 | 4 | 1 | 2 | 1 | 1 | 1 | 1 |  |
| ***H. indicus*** | 2 | 2 | 2 | 2 | 2 | 1 | 1 | 1 | 2 | 1 | 2 | 2 | 2 | 2 | 2 | 1 | 1 | 3 | 1 | 3 | 5 | 1 | 1 | 2 | 2 | 2 | 4 | 3 | 2 | 1 | 2 | 1 | 1 |  |
| ***H. pubescens*** | 2 | 2 | 1 | 2 | 2 | 1 | 1 | 1 | 2 | 2 | 1 | 2 | 3 | 1 | 2 | 1 | 2 | 3 | 1 | 2 | 4 | 2 | 1 | 1 | 1 | 1 | 4 | 3 | 2 | 1 | 1 | 2 | 1 |  |
| ***I. frutescens*** | 2 | 2 | 2 | 2 | 2 | 2 | 1 | 1 | 1 | 2 | 1 | 1 | 1 | 1 | 2 | 1 | 2 | 3 | 1 | 3 | 5 | 2 | 1 | 2 | 1 | 1 | 4 | 5 | 1 | 1 | 2 | 1 | 1 |  |
| ***M. tenacissima*** | 1 | 1 | 2 | 2 | 2 | 1 | 2 | 2 | 3 | 1 | 2 | 2 | 2 | 2 | 1 | 2 | 2 | 3 | 2 | 3 | 5 | 1 | 1 | 2 | 2 | 2 | 4 | 5 | 3 | 2 | 1 | 2 | 1 |  |
| ***N. oleander*** | 2 | 2 | 2 | 2 | 2 | 1 | 1 | 1 | 2 | 2 | 1 | 2 | 5 | 1 | 2 | 1 | 2 | 3 | 1 | 1 | 3 | 3 | 1 | 1 | 1 | 1 | 2 | 3 | 2 | 3 | 1 | 1 | 1 |  |
| ***R. serpentina*** | 2 | 2 | 2 | 1 | 2 | 2 | 2 | 2 | 2 | 2 | 1 | 2 | 5 | 1 | 2 | 1 | 1 | 2 | 3 | 2 | 7 | 1 | 1 | 3 | 2 | 2 | 4 | 4 | 3 | 1 | 1 | 1 | 2 |  |
| ***T. divaricata*** | 2 | 1 | 2 | 1 | 2 | 1 | 2 | 2 | 2 | 2 | 1 | 4 | 5 | 2 | 2 | 1 | 2 | 2 | 3 | 1 | 7 | 2 | 1 | 3 | 1 | 2 | 3 | 5 | 2 | 3 | 1 | 1 | 1 |  |

**Additional file:** NMR spectroscopic data of all the identified marker compounds from the crude root extracts of fourteen species of family Apocynaceae.

***Asclepias currasavica***

**Compound 1: Pekilocerin A** (Ahmad and Basha, 2010)

**^1^H NMR** (C_5_D_5_N, 400 MHz) : δ 10.0 (1H, s, H-19), 6.10 (1H, br s, H-22), 5.24 (1H, dd, J=18.0, 1.0 H-21b), 5.01 (1H, s, H-1 of sugar), 4.99 (1H, dd, J=18.0, 1.0 Hz, H-21a), 4.46 (1H, td, J=12.0, 4.0 Hz, H-3), 4.32 (1H, td, J=12.0, 4.0 Hz, H-2), 4.12 (1H, dd, J=12.0, 5.0 Hz, H-3 of sugar), 3.76 (1H, m, H-5 of sugar), 2.74 (1H, dd, J=9.0, 5.0 Hz, H-17), 2.48 (1H, dd, J=12.0, 4.0 Hz, H-1b), 2.12 (1H, q, J=12.0 Hz, H-4B of sugar), 2.02 (1H, td, J=5.0 12.0 Hz, H-4A of sugar), 1.74 (1H, td, J=4.0 12.0 Hz, H-4a), 1.59 (1H, q, J=12.0 Hz, H-4b), 1.37 (1H, d, J=6.0 Hz, H-6 of sugar), 1.16 (1H, t, J=12.0 Hz, H-1a), 0.90 (3H, s, H-18).

**^13^C NMR** (C_5_D_5_N, 100 MHz)2 : δ 207.8 (C-19), 175.4 (C-20), 174.3 (C-23), 117.8 (C-22), 72.3 (C-3), 69.3 (C-2), 84.0 (C-14), 73.6 (C-21), 52.8 (C-10), 51.2 (C-17), 49.7 (C-13),48.7 (C-9), 43.4 (C-8), 42.5 (C-5), 39.2 (C-12), 36.5 (C-1), 33.9 (C-15), 32.5 (C-4), 27.9 (C-7), 27.9 (C-6), 27.1 (C-16), 22.2 (C-11), 15.2 (C-18), Sugar 97.2 (C-1), 92.7 (C-2), 73.8 (C-3), 68.5 (C-5), 39.9 (C-4), 21.5 (C-6).

**HR-MS *m/z*** 550.2778 [M+H_2_O]^+^ (calcd. For C_29_H_42_O_10_, 550.2778).

**Compound 2: Uzarin** (Ahmad and Basha, 2010)

**^1^H NMR** (C_5_D_5_N, 500 MHz) : δ 0.66 (3H, s, H-19), 0.75 (H, m, H -9a), 0.76 (H, m, H-1a), 0.79 (H, m, H -5a), 0.93 (3H, s, H-18), 1.04 (H, m, H-6a), 1.04 (H, m, H-7a), 1.09 (H, m, H-11b), 1.18 (H, m, H-6b), 1.19 (H, m, H-12a), 1.30 (H, m, H-11a), 1.30 (H, m, H-12b), 1.51 (H, m, H-4a), 1.53 (H, m, H-1b), 1.59 (H, m, H-8b), 1.66 (H, m, H-2b), 1.79 (H, m, H-15b), 1.90 (H, m, H-16b), 1.91 (H, m, H-4b), 1.94 (H, m, H-15a), 2.02 (H, m, H-16a), 2.06 (H, m, H-2a), 2.21 (H, m, H-7b), 2.71 (H, m, H-17a), 3.90 (H, m, H-3a), 3.90 (H, m, H-5 of Glc I), 3.90 (H, m, H-5 of Glc II), 4.05 (H, m, H-2 of Glc II), 4.09 (H, m, H-2 of Glc I), 4.13 (H, m, H-4 of Glc I), 4.19 (H, m, H-3 of Glc II), 4.24 (H, m, H-4 of Glc II), 4.30 (H, m, H-6A of Glc I), 4.32 (H, m, H-3 of Glc I), 4.37 (H, m, H-6A of Glc II), 4.46 (H, m, H-6B of Glc II), 4.50 (H, m, H-6b of Glc I), 4.97 (H, m, H-21a), 5.02 (H, m, H-1 of Glc I), 5.20 (H, m, H-1 of Glc II), 5.25 (H, m, H-21b), 6.07 (H, m, H-22).

**^13^C NMR** (C_5_D_5_N, 125 MHz): δ 176.1 (C-20), 174.6 (C-23), 117.4 (C-22), 84.2 (C-14), 78.1 (C-3), 73.7 (C-21), 51.2 (C-17), 49.7 (C-9), 49.9 (C-13), 44.2 (C-5), 41.1 (C-8), 39.5 (C-12), 37.2 (C-1), 35.8 (C-10), 34.5 (C-4), 32.9 (C-15), 29.7 (C-2), 28.8 (C-6), 27.8 (C-7), 27.1 (C-16), 21.3 (C-11), 16.0 (C-18), 12.1 (C-19), Glc I 101.0 (C-1), 84.4 (C-2), 78.4 (C-5), 77.7 (C-3), 71.3 (C-4), 62.4 (C-6), Glc II 106.2 (C-1), 76.7 (C-2), 77.8 (C-3), 71.3 (C-4), 78.5 (C-5), 62.4 (C-6).

**HR-MS *m/z*** 699.3592 **[M+H]^+^** (calcd. For C_35_H_55_O_14_, 699.3592).

***Calotropis gigentea***

**Compound 1: Calotropone** (Wang et al. 2008)

**^1^H NMR** (400 MHz, CDCl_3_) δ 7.93 (2H, d, 7.5 Hz, H-2’,6’), 7.56 (1H, t, 7.5 Hz, H-4’), 7.43 (2H, t, 7.5 Hz, H-3’,5’), 5.41 (1H, m, H-6), 4.80 (1H, dd, 11.3, 4.5 Hz, H-12), 3.53 (1H, m, H-3), 2.90 (1H, m, H-16a, H-16a), 2.33 (1H, dd, 12.8, 3.6 Hz, H-4a), 2.25 (1H, m, overlapped, H-4b), 2.20 (1H, m, H-7 ), 2.12 (1H, m, H-15a), 2.06 (3H, overlapped, H-11), 1.92-1.75 (6H, m, H-1a, 2a, 7b, 8, 15b, 16b), 1.50-1.32 (3H, m, H-2b, 9, 11a, 18, ), 1.13 (1H, m, H-1b), 0.98 (3H, H-19).

**^13^C NMR** (100 MHz, CDCl_3_) δ 209.3 (C-20), 165.3 (C-7’), 139.5 (C-5’), 133.2 (C-4’), 129.9 (C-1’), 129.5 (C-3’,5’), 128.4 (C-2’,6’), 121.1 (C-6), 91.2 (C-17), 88.5 (C-14), 73.1 (C-12), 71.4 (C-3), 57.5 (C-13), 42.6 (C-9), 41.9 (C-4), 37.0 (C-1,8), 36.7 (C-10), 31.8 (C-16), 31.7 (C-15), 31.4 (C-2), 27.4 (C-21), 26.5 (C-11), 26.0 (C-7), 19.4 (C-19), 7.7 (C-18).

**HR-MS *m/z*** 507.2149 **[M+K]^+^** (calcd. For C_28_H_36_O_6_K, 507.2149).

**Compound 2: Calactinic acid; 3'-Et ester** (Roy et al. 2005, Seeka and Sutthivaiyakit 2010)

**^1^H NMR** (400 MHz, CDCl_3_-C_5_D_5_N, 9:1) δ 9.79 (1H, s, H-19), 5.73 (1H, s, H-22), 4.90 (1H, dd, *J*=18.0, 2.0 Hz, H-21a), 4.88 (1H, s, H-1′), 4.67 (1H, dd, *J*=18.0, 2.0 Hz, H-21b), 4.42 (1H, m, H-5′), 3.58 (3H, s, COOCH_3_-3′), 3.31 (1H, ddd, *J*=13.0, 12.0, 5.0 Hz, H-2), 3.14 (1H, ddd, *J*=13.0, 12.0, 5.0 Hz, H-3), 2.62 (1H, dd, *J*=9.6, 4.8 Hz, H-17), 2.50 (1H, dd, *J*=13.0, 5.0 Hz, H-1a), 2.21 (1H, dd, *J*=13.0, 10.0 Hz, H-4′a), 2.19 (1H, m, H-6a), 2.06 (1H, dd, *J*=13.0, 5.6 Hz, H-4′b), 1.97 (1H, m, H-16a), 1.87 (1H, m, H-15a), 1.75 (1H, m, H-16b), 1.59 (2H, m, H-15b, H-7a), 1.50 (2H, m, H-8, H-4a), 1.30 (1H, m, H-12a), 1.24 (3H, d, *J*=6.0 Hz, H-6′), 1.20 (1H, m, H-12b), 1.17 (1H, m, H-9), 1.14 (1H, m, H-5), 1.11 (1H, m, H-4b), 1.10 (2H, m, H-6b, H-7b), 0.83 (1H, t, *J*=13.0 Hz, H-1b), 0.69 (3H, s, H-18).

**^13^C NMR** (100 MHz, CDCl_3_-C_5_D_5_N, 9:1) δ 207.1 (C-19), 174.5 (C-20), 174.4 (C-23), 171.2 (C-3′), 117.3 (C-22), 108.5 (C-1′), 85.2 (C-3), 84.1 (C-2′), 83.8 (C-14), 76.1 (C-5′), 73.1 (C-21), 70.0 (C-2), 51.8 (C-10), 51.5 (COOCH3-3′C), 50.4 (C-17), 49.2 (C-13), 48.0 (C-5), 42.3 (C-9), 41.8 (C-8), 40.2 (C-4′), 38.9 (C-12), 37.9 (C-1), 34.0 (C-4), 31.7 (C-15), 27.3 (C-11), 26.9 (C-6), 26.4 (C-16), 21.8 (C-6′), 21.5 (C-7), 15.4 (C-18).

**HR-MS *m/z*** 599.2832 [M +Na]^+^ (calcd for C_30_H_43_O_10_Na, 599.2832).

***Calotropis procera***

**Compound 1: Proceraside A** (Ibrahim et al, 2014)

**^1^H NMR** (DMSO-d_6_, 400 MHz): δ H 2.01 (1H, m, H-1a), 1.78 (1H, m, H-1b), 2.15 (1H, m, H-2A), 1.84 (1H, m, H-2B), 3.53 (1H, m, H-3), 1.81 (1H, m, H-4a), 1.09 (1H, m, H-4b), 1.45 (1H, m, H-5), 2.26 (1H, m, H-6a), 1.53 (1H, m, H-6b), 2.13 (1H, m, H-7a), 1.84 (1H, m, H-7b), 1.42 (1H, m, H-8), 1.22 (1H, m, H-9), 1.62 (2H, m, H-11), 1.60 (1H, m, H-12a), 1.39 (1H, m, H-12b), 1.91 (1H, m, H-15a), 1.53 (1H, m, H-15b), 2.02 (1H, m, H-16a), 1.81 (1H, m, H-16b), 2.71 (1H, dd, *J*=9.8, 6.0 Hz, H-17), 0.69 (3H, s, H-18), 9.83 (1H, s, H-19), 4.95 (1H, br d, *J*=18.0 Hz, H-21a), 4.86 (1H, br d, *J*=18.0 Hz, H-21b), 5.90 (1H, s, H-22), 4.50 (1H, d, *J*=8.0 Hz, H-1´), 4.04 (1H, dd, *J*=8.0, 2.8 Hz, H-2´), 3.77 (1H, m, H-3´), 3.14 (1H, dd, *J*=8.8, 2.8 Hz, H-4´), 3.51 (1H, dq, *J*=8.5, 6.3 Hz, H-5´), 1.19 (3H, d, *J*=6.3 Hz, H-6´), 1.98 (3H, s, 20 -CH_3_CO).

**^13^C NMR** (DMSO-d_6_, 100 MHz): δ 209.4 (C-19), 176.1 (C-20), 174.5 (C-23), 173.7 (20 -CH_3_CO), 116.2 (C-22), 98.1 (C-1´), 83.2 (C-14), 75.0 (C-2´), 75.7 (C-3), 73.0 (C-21), 73.6 (C-4´), 72.6 (C-3´), 70.5 (C-5´), 55.9 (C-10), 51.1 (C-13), 49.9 (C-17), 47.4 (C-9), 42.5 (C-5), 41.8 (C-8), 38.8 (C-12), 35.5 (C-4), 31.4 (C-15), 30.2 (C-1), 27.9 (C-2), 27.8 (C-6), 27.3 (C-7), 26.0 (C-16), 21.2 (C-11), 21.0 (20 -CH_3_CO), 18.5 (C-6´), 15.4 (C-18).

**HR-MS *m/z*** 577.3013 [M+H]^+^ (calcd for C_31_H_45_O_10_, 577.3013).

**Compound 2: Calotropagenin** (Seeka and Sutthivaiyakit, 2010)

**^1^H NMR** (CDCl_3_): δ 5.18 (1H, dd, *J*=9.9, 8.1 Hz, H-12), 4.51 (1H, dd, *J*=8.7, 5.6 Hz, H-3a), 1.06 (3H, brs, H-23), 1.01 (3H, brs, H-27), 0.97 (3H, brs, H-24), 0.95 (3H, d, *J*=6.5 Hz, H-30), 0.87 (3H, brs, H-28), 0.83 (3H, d, *J*=6.6 Hz, H-29), 0.79 (3H, brs, H-26);

**^13^C NMR** (CDCl3): δ 170.15 (C-25), 138.59 (C-13), 124.34 (C-12), 80.63 (C-3), 59.06 (C-18), 55.26 (C-5), 47.63 (C-9), 43.29 (C-10), 41.53 (C-14), 41.33 (C-22), 39.86 (C-20), 39.62 (C-8), 39.62 (C-19), 38.44 (C-4), 37.71 (C-1), 33.16 (C17), 32.95 (C-7), 31.23 (C-21), 29.67 (C-15), 29.09 (C-2), 28.73 (C-23), 26.60 (C-28), 25.79 (C-16), 23.65 (C-27), 22.45 (C-11), 21.37 (C-30), 18.24 (C-6), 17.49 (C-26), 16.84 (C-29), 15.69 (C-24).

**HR-MS *m/z*** 439.3576 [M+H]^+^ (calcd for C_30_H_47_O_2_, 439.3576).

***Carissa carandas***

**Compound 1: Carandinol** (Begum et al. 2013)

**^1^H NMR** (400 MHz, CDCl_3_) δ 3.18 (1H, dd, J=11, 4.5 Hz, H-3), 1.86 (1H, m, H-20a), 1.70-1.68 (2H, m, H-22, 1a), 1.61-1.59 (2H, m, H-20b, 19a), 1.55-1.52 (2H, m, H-2a, 6a), 1.50-1.45 (3H, m, H-7a, 11a, 12a), 1.42-1.37 (5H, m, H-16a, 15a, 13, 12b, 6b), 1.29-1.26 (2H, m, H-11b, 2b), 1.23-1.18 (3H, m, H-16b, 15b, 7b), 1.02 (1H, dd, J=11.0, 3.0, H-17), 0.91 (1H, m, H-1b), 0.95- 0.92 (12H, m, H-23, 26, 27, 30), 0.89-0.86 (6H, brs, H-28, 29), 0.84 (1H, m, H-19b), 0.79 (3H, s, H-25), 0.74 (3H, s, H-24), 0.66 (1H, dd, J=11, 1.5 Hz, H-5).

**^13^C NMR** (100 MHz, CDCl_3_) δ 83.8 (C-21), 79.0 (C-3), 56.5 (C-17), 55.0 (C-5), 50.3 (C-9), 49.5 (C-13), 44.8 (C-18), 42.4 (C-14), 41.8 (C-8), 40.9 (C-19), 38.9 (C-4), 38.7 (C-1), 37.1 (C-10), 36.9 (C-22), 34.9 (C-20), 33.3 (C-7), 33.0 (C-15), 28.0 (C-23), 27.4 (C-2), 23.4 (C-12), 21.0 (C-11), 18.6 (C-29), 18.4 (C-16), 18.2 (C-6), 17.6 (C-30), 16.8 (C-26,29), 16.3 (C-27), 15.9 (C-25), 15.4 (C-24).

**HR-MS *m/z*** 483.3604 [M+K]^+^ (calcd. For C_30_H_52_O_2_K, 483.3604).

***Carissa spinarum***

**Compound 1: Cycloolivil** (wahab Sab et al. 2015)

**^1^H NMR** (400 MHz, CD_3_OD) δ 6.77 (1H, d, J = 8.0 Hz, H‑5′), 6.70 (1H, d, J = 8.0 Hz, H‑2′), 6.67 (1H, dd, J = 8.0, 2.0, H‑6′), 6.63 (1H, s, H‑8), 6.18 (1H, s, H‑5), 4.03 (1H, d, J = 11.6 Hz, H‑4), 3.81 (1H, d, J = 2.4 Hz, H‑3a), 3.80 (3H, s, ‑OCH3 ), 3.79 (1H, d, J = 11.2 Hz, H‑2a), 3.75 (3H, s, ‑OCH3 ), 3.58 (1H, d, J=4.4 Hz, H‑2b), 3.55 (1H, d, J = 4.4 Hz, H‑3b), 3.26 (1H, d, J = 16.8, H‑1b ), 2.63 (1H, d, J = 16.8, H‑1a ), 2.05 (1H, d, J = 2.8, H‑3).

**^13^C NMR** (100 MHz, CD_3_OD) δ 149.28 (C‑3′), 147.65 (C‑7), 146.26 (C‑4′), 145.46 (C‑6), 138.63 (C‑1′), 133.71, (C‑10), 126.59 (C‑9), 123.72 (C‑6′), 117.50 (C‑5), 116.17 (C‑5′), 114.10 (C‑2′), 113.13 (C‑8), 75.11 (C‑2), 69.57 (C‑2), 61.00 (C‑3), 56.55 (‑OCH_3_), 56.52 (‑OCH_3_), 47.73 (C‑3), 45.05 (C‑4), 40.08 (C‑1).

**HR-MS *m/z*** 399.1420 [M+Na]^+^ (calculated for C_20_H_24_NaO_7,_ 399.1420).

***Catharanthus roseus***

**Compound 1:** Ajmaline (Itoh et al. 2005)

**^1^H NMR** (400 MHz, CDCl_3_) δ 7.54 (1H, dd, *J*=7.5, 1.0 Hz, H-9), 7.16 (1H, td, *J*=7.5, 1.0 Hz, H-11), 6.82 (1H, td, *J*=7.5, 1.0 Hz, 10), 6.76 (1H, brd, *J*=7.5 Hz, 12), 4.42 (1H, brs, H-17), 3.94 (1H, brd, *J*=9.5 Hz, H-3), 3.73 (1H, m, H-5), 2.83 (1H, brs, H-2), 2.79 (3H, s, N_a_Me), 2.59 (1H, brtd, *J*=5.5, 1.0 Hz, H-16), 2.54 (1H, brdd, H-15), 2.35 (1H, brd, *J*=14.5 Hz, H-6a), 2.27-2.25 (2H, m, H-6b, 14b), 1.98 (1H, m, H-20), 1.95 (1H, brdd, *J*=14.5, 5.5 Hz, H-14a), 1.72-1.56 (2H, m, H-19a, 19b), 1.07 (t, *J*=7.5 Hz, 3H).

**^13^C NMR** (100 MHz, CDCl_3_) δ 154.4 (C-13), 132.6 (C-8), 129.2 (C-11), 124.7 (C-9), 121.6 (C-10), 111.3 (C-12), 98.4 (C-21), 78.6 (C-2), 76.7 (C-17), 64.6 (C-5), 54.2 (C-3), 51.1 (C-20), 34.9 (C- N_a_Me), 32.1 (C-6), 30.9 (C-14), 28.3 (C-15), 26.1 (C-19), 12.1 (C-18).

**HR-MS *m/z*** 327.4 **[M+H]^+^** (calcd. For C_20_H_27_N_2_O_2_, 327.4).

**Compound 2: Cadin-2-en-1β-ol-1β-D-glucuronopyranoside** (Chung et al. 2007)

**^1^H NMR** (MeOD, 500 MHz) δ: 6.84 (d, J＝9.5 Hz, 1H, H-2), 4.11 (br s, 1H, H-1'), 3.92 (d, J＝11.0 Hz, 1H, H-5'), 3.89 (dd, J＝9.5, 4.5 Hz, 1H, H-1α), 3.85 (d, J＝4.5 Hz, 1H, H-2'), 3.75—3.77 (m, 1H, H-3'), 3.65—3.68 (m, 1H, H-4'), 2.46 (d, J＝7.5 Hz, 1H, H-4a), 2.29 (d, J＝9.5 Hz, 1H, H-4b), 2.04 (br s, 3H, H3-11), 1.60—1.63 (m, 1H, H-5), 1.58—1.61 (m, 1H, H-6α), 1.32 (dd, J＝4.5, 7.0 Hz, 1H, H-10α), 1.25 (br s, 4H, H2-7, H2-8), 1.04—1.07 (m, 1H, H-9), 0.95 (d, J＝7.55 Hz, 3H, Me-15), 0.92 (d, J＝7.1 Hz, 3H, Me-13), 0.89 (d, J＝6.5 Hz, 3H, Me-14).

**^13^C NMR** (MeOD, 125 MHz) δ: 147.21 (C-3), 119.61 (C-2), 70.78 (C-1), 56.59 (C-10), 56.48 (C-5), 56.20 (C-9), 51.08 (C-6), 34.72 (C-12), 32.14 (C-11), 29.57 (C-7), 29.48 (C-8), 25.33 (C-4), 22.90 (C-14), 21.22 (C-15), 19.33 (C-13), GlcUA 179.13 (C-6'), 103.06 (C-1'), 76.15 (C-5'), 68.13 (C-2'), 66.23 (C-3'), 64.58 (C-4').

**HR-MS *m/z*** 399.2383 **[M+H]^+^** (calcd. For C_21_H_35_O_7_, 399.2383).

***Cryptolepis dubia***

**Compound 1: Cryptanoside A** (Ahmad and Basha, 2010)

**^1^H NMR** (400 MHz, CDCl_3_) δ 5.99 (1H, dt, *J*=1.0, 1.8 Hz, H-22), 4.93 (1H, br d, *J*=2.4 Hz, H-1 of Ole), 4.82 (2H, H-21), 4.69 (1H, dd, *J*=12.7, 4.1 Hz, H-11), 3.90 (1H, t, *J*=8.3 Hz, H-17), 3.82 (1H, br s, H-3), 3.68 (1H, dq, *J*=9.3, 6.3 Hz, H-5 of Ole), 3.63 (1H, d, *J*=4.1 Hz, 11-OH), 3.51 (1H, ddd, *J*=11.1, 9.2, 5.0 Hz, H-3 of Ole), 3.40 (3H, 3-OCH3 of Ole), 3.36 (1H, d, *J*=6.3 Hz, H-7), 3.14 (1H, t, *J*=9.1 Hz, H-4), 2.69 (1H, d, *J*=2.1 Hz, 14-OH), 2.34 (1H, dd, *J*=15.9, 6.3 Hz, H-6a), 2.21 (1H, d, *J*=12.7 Hz, H-9), 1.24 (3H, d, *J*=6.4 Hz, H-6 of Ole), 1.22 (3H, s, H-20), 1.05 (3H, s, H-19).

**^13^C NMR** (100 MHz, CDCl_3_) δ 213.0 (C-12), 173.4 (C-23), 170.5 (C-20), 118.8 (C-22), 81.2 (C-14), 73.7 (C-11), 73.7 (C-21), 70.5 (C-3), 63.2 (C-8), 63.2 (C-13), 52.5 (C-7), 42.4 (C-17), 35.8 (C-6), 35.7 (C-5), 34.7 (C-9), 34.7 (C-10), 32.6 (C-4), 32.5 (C-1), 28.5 (C-15), 27.0 (C-16), 26.9 (C-2), 23.2 (C-19), 18.4 (C-18), Ole 95.5 (C-1), 78.3 (C-3), 76.2 (C-4), 67.7 (C-5), 56.4 (OCH3), 34.5 (C-2), 17.8 (C-6).

**HR-MS *m/z*** 601.1778 **[M+K]^+^** (calcd. For C_30_H_42_O_10_K, 601.1778).

***Hemidesmus indicus***

**Compound 1: Denicunine** (Ahmad and Basha, 2010)

**^1^H NMR** (400 MHz, CDCl_3_) δ 5.38 (1H, m, H-6), 4.62 (1H, dd, *J*=8.0 Hz, 2.0 Hz, H-1 of Ole), 4.30 (1H, d, *J*=8.0 Hz, H-1 of Dil), 3.88-3.84 (2H, m, H-5 of Ole and Dil), 3.78-3.74 (1H, m, H-20), 3.62-3.56 (2H, m, H-3 of Ole and Dil), 3.52 (3H, s, OCH_3_ of Ole), 3.48-3.42 (1H, m, H-4 of Dil), 3.40 (3H, s, OCH_3_ of Dil), 3.30 (1H, t, *J*=8.0 Hz, H-2 of Dil), 3.12 (H, t, *J*=9.0 Hz, H-4 of Ole), 2.24-2.16 (H, m, H-2eq of Ole), 1.70-1.58 (H, m, H-2a of Ole), 1.35 (3H, d, *J*=6.0 Hz, H-6 of Ole), 1.32 (3H, d, *J*=6.0 Hz, H-6 of Dil), 1.29 (3H, d, *J*=6.0 Hz, H-21), 1.091 (3H, s, H-18), 0.74 (3H, s, H-19).

**^13^C NMR** (100 MHz, CDCl_3_) δ 139.0 (C-5), 122.5 (C-6), 86.3 (C-14), 79.3 (C-3), 66.1 (C-20), 55.0 (C-17), 48.2 (C-9), 44.3 (C-13), 39.6 (C-4), 36.1 (C-12), 37.1 (C-10), 35.9 (C-1), 30.0 (C-15), 30.4 (C-8), 27.9 (C-2), 27.9 (C-7), 27.7 (C-16), 24.8 (C-11), 22.5 (C-21), 17.9 (C-19), 15.4 (C-18), Ole 102.9 (C-1), 75.0 (C-3), 74.3 (C-2), 74.1 (C-4), 69.6 (C-5), 61.2 (OCH3), 20.7 (C-6), Dil 102.2 (C-1), 83.2 (C-4), 78.3 (C-3), 70.0 (C-5), 57.1 (OCH3), 37.5 (C-2), 19.3 (C-6).

**HR-MS *m/z*** 679.2564 [M+ ACN]^+^ (calcd. For C_37_H_61_O_10_N, 679.2564).

**Compound 2: Emidine** (Ahmad and Basha, 2010)

**^1^H NMR** (C_5_D_5_N, 400 MHz) : δ 5.32-5.38 (1H, m, H-6), 4.62 (1H, dd, *J*=9.0, 2.0 Hz, H-1 of Dig I), 4.50 (1H, d, *J*=9.0, 2.0 Hz, H-1 of Dix II), 4.43 (1H, dd, *J*=9.0, 2.0 Hz, H-1 of Dix III), 3.82-3.90 (1H, m, H-20), 3.72-3.76 (2H, m, H-4 of Dix I, H-4 of Dix II), 3.64-3.68 (3H, m, H-3 of Dix I, H-3 of Dix II, H-3 of Dix III), 3.44-3.48 (3H, m, H-5 of Dix I, H-5 of Dix II, H-5 of Dix III), 3.36-3.40 (1H, m, H4 of Dix III), 2.02-2.33 (3H, m, H-2eq of Dix I, H-2eq of Dix II, H-2eq of Dix III), 1.78-2.02 (1H, m, H-2a of Dix I, H-2a of Dix II, H-2ax of Dix III), 1.34 (3H, d, *J*=6.0 Hz, H-6 of Dix I), 1.26 (3H, d, *J*=6.0 Hz, H-21), 1.20 (3H, d, *J*=6.0 Hz, H-6 of Dig II), 1.18 (3H, d, *J*=6.0 Hz, H-6 of Dix III), 1.02 (3H, s, H-18), 0.89 (3H, s, H-19).

**^13^C NMR** (CDCl_3_, 100 MHz) : δ 141.8 (C-5), 122.5 (C-6), 87.2 (C-17), 84.9 (C-14), 77.4 (C-3), 75.3 (C-8), 71.5 (C-12), 71.5 (C-20), 56.0 (C-13), 45.8 (C-9), 40.4 (C-1), 39.5 (C-4), 37.9 (C-10), 32.5 (C-15), 31.7 (C-16), 32.5 (C-7), 30.3 (C-2), 24.4 (C-11), 18.8 (C-19), 15.5 (C-21), 12.2 (C-18), Ole 97.0 (C-1), 83.9 (C-4), 67.3 (C-3), 67.3 (C-5), 38.7 (C-20), 18.8 (C-6), Dix 106.7 (C-1), 78.6 (C-5), 77.3 (C-3), 75.3 (C-2), 72.1 (C-4), 63.1 (C-6), Glc 101.9 (C-1), 83.2 (C-4), 80.0 (C-3), 72.8 (C-5), 57.3 (OCH3), 37.4 (C-2), 18.0 (C-6).

**HR-MS *m/z*** 765.4398 [M+ ACN]^+^ (calcd. For C_41_H_67_O_12_N, 765.4398).

***Hollarhena pubescence***

**Compound 1: Holonamine** (Nnadi et al. 2017)

**^1^H-NMR** (600 MHz CDCl_3_; δ 7.86 (1H, d, *J*=10.3 Hz, H-4), 7.48 (1H, d, *J*=3.0 Hz, H-18), 6.15 (1H, dd, *J*= 10.3, 2.0 Hz, H-2), 6.10 (1H, t, *J*= 1.6 Hz, H-1), 4.05–4.10 (2H, m, H-11, H-17),), 2.51 (1H, tdd, *J*= 13.5, 5.1, 1.5 Hz, H-6a), 2.39 (1H, ddd, *J*= 13.2, 4.4, 2.5 Hz, H-6b), 2.19 (1H, dd, *J*= 12.2, 4.8 Hz, H-12a), 2.10 (1H, m, H-20), 2.06 (1H, m, H-7a), 1.91 (1H, qd, *J*= 11.4, 3.8 Hz, H-8), 1.78 (1H, ddd, *J*= 14.0, 8.2, 2.8 Hz, H-15a), 1.68 (1H, m, H-16a), 1.63 (1H, dd, *J*= 12.2, 4.8 Hz, H-12b), 1.45 (1H, ddd, *J*= 14.2, 8.5, 3.0 Hz, H-15b), 1.38 (1H, td, *J*= 11.5, 5.3 Hz, H-14), 1.36 (3H, s, H-19), 1.35 (3H, d, H-21), 1.32 (1H, m, H-9), 1.15 (1H, ddd, *J*= 12.8, 5.0, 3.8 Hz, H-7b), 0,83 (1H, m, H-16b).

**^13^C NMR** (100 MHz, CDCl_3_) δ 186.92 (C-3), 168.37 (C-18), 168.05 (C-5), 159.10 (C-4), 125.27 (C-2), 124.78 (C-1), 68.91 (C-11), 68.75 (C-20), 66.37 (C-13), 59.94 (C-9), 53.40 (C-14), 48.18 (C-17), 44.21 (C-12), 43.85 (C-10), 36.27 (C-8), 34.19 (C-7), 33.10 (C-6), 29.26 (C-16), 24.39 (C-15), 18.72 (C-19), 16.96 (C-21).

**HR-MS *m/z*** 326.2000 [M + H]+ ; calculated for [C_21_H_28_NO_2_]^+^ : 326.2120.

**Compound 2: Conessimine** (Nnadi et al. 2017)

**^1^H-NMR** (600 MHz CDCl3; δ: 5.35 (1H, dt, 5.1, 1.8, H-6), 3.19 (1H, qd, 6.5, 4.7, H-20), 2.79 (1H, d, 12.1, H-18b), 2.52 (1H, d, 12.1, H-18a), 2.27 (6H, s, H-22, H-23), 2.19 (2H, m, H-4), 2.10 (1H, m, H-3), 2.08 (1H, m, H-7a), 2.05 (1H, m, H-7b), 1.94 (1H, m, H-1a), 1.89 (1H, m, H-17), 1.88 (1H, m, H-12a), 1.78 (1H, m, H-2a), 1.69 (1H, m, H-16a), 1.69 (1H, m, H-11a), 1.64 (1H, m, H-15a), 1.51 (1H, m, H-15b), 1.46 (1H, m, H-2b), 1.41 (1H, m, H-12b), 1.32 (1H, m, H-8), 1.31 (1H, m, H-14), 1.23 (1H, m, H-11b), 1.16 (1H, m, H-16b), 1.12 (3H, d, 6.6, H-21), 1.08 (1H, m, H-1b), 0.99 (1H, m, H-9), 0.93 (3H, s, H-19).

**^13^C NMR** (100 MHz, CDCl_3_) δ 142.56 (C-5), 122.05 (C-6), 66.33 (C-3), 57.30 (C-20), 56.89 (C-14), 56.10 (C-17), 55.11 (C-13), 52.76 (C-18), 51.35 (C-9), 41.78 (C-22), 41.78 (C-23), 39.44 (C-1), 38.37 (C-12), 37.93 (C-10), 36.06 (C-4), 34.94 (C-8), 33.12 (C-7), 28.11 (C-16), 25.63 (C-2), 23.64 (C-15), 23.11 (C-11), 19.88 (C-19), 15.93 (C-21).

**HR-MS *m/z*** 343.4082 [M + H]+ ; calculated for [C_23_H_39_N_2_]^+^ : 343.3113.

**Compound 3: Regholarrhemine D** (Bhutani et al. 1990)

**^1^HNMR:** δ 5.36 (s, lH, C-6), 4.80 (s, 1H, D_2_0 exchangeable N-OH), 2.90 (d, 2H, *J* =8 Hz, H-3 a and C-20a H), 2.67 (m, 2H, H-18), 2.34 (s, 6H, NMe_2_), 1.34 (d, 3H, *J*=7 Hz), 0.96 (3H, s, H-19).

**^13^C NMR:** (100 MHz, CDCl_3_) δ 141.8 (C-5), 120.3 (C-6), 64.8 (C-3,18), 56.5 (C-20), 55.3 (C-14), 53.1 (C-17), 50.5 (C-13), 49.8 (C-9), 41.6 (C-22,23), 38.3 (C-1), 37.8 (C-10), 36.5 (C-16), 35.3 (C-4), 33.7 (C-8), 31.8 (C-7), 326.7 (C-12), 24.9 (C-2), 22.5 (C-15), 21.8 (C-11), 19.3 (C-19), 14.8 (C-21).

**HR-MS *m/z*** 359.3062 [M+H]^+^ (calcd for C_23_H_39_N_2_O, 359.3062).

***Ichnocarpus frutescens***

**Compound 1: Octyl tetracontane** (Aggarwal et al. 2010)

**^1^H NMR** (400 MHz, CDCl_3_) δ: 2.26 (2H, m, CH_2_), 2.04 (2H, m, CH_2_), 1.54 (4H, m, 2 CH_2_), 1.25 (86H, brs, 43 CH_2_), 0.87 (3H, t, *J*=6.8 Hz, H-1), 0.84 (3H, t, *J*=6.3 Hz, H-48).

**^13^C NMR (100 MHz, CDCl_3_) δ:** 31.90 (CH_2_), 29.67 (39 CH_2_), 29.63 (CH_2_), 28.59 (CH_2_), 27.36 (CH_2_), 26.15 (CH_2_), 25.89 (CH_2_), 22.67 (CH_2_), 14.09 (C-1, C-48).

**HR-MS *m/z*** 698.3018 [M+Na]^+^ (calcd. For C_48_H_98_Na, 698.3018).

**Compound 2: 20-(2-Hydroxyphenyl) eicosyl eicosanoate** (Aggarwal et al. 2010)

**^1^H NMR** (400 MHz, CDCl_3_) δ: 7.52 (1H, d, *J*=9.0 Hz, H-23´), 7.35 (1H, m, H-24´), 7.13 (1H, dd, *J*=8.7, 2.1 Hz, H-26´), 7.06 (1H, m, H-25´), 4.13 (1H, d, *J*=7.2 Hz, H-1´a), 4.08 (1H, d, *J*=6.6 Hz, H-1´b), 2.82 (1H, d, *J*=7.2 Hz, H-2a), 2.78 (1H, d, *J*=7.5 Hz, H-2b), 2.68 (1H, d, *J*=7.2 Hz, H-20´a), 2.58 (1H, d, *J*=7.2 Hz, H-20´b), 2.30 (2H, m, CH_2_), 2.04 (2H, m, CH_2_), 1.68 (2H, m, CH_2_), 1.60 (2H, m, CH_2_), 1.42 (4H, m, 2 CH_2_), 1.37 (4H, br s, 2 CH_2_), 1.33 (6H, br s, 3 CH_2_), 1.28 (24H, br s, 12 CH_2_), 1.25 (22H, br s, 11 CH_2_), 0.85 (3H, t, *J*=6.3 Hz, H-20).

**^13^C NMR (100 MHz, CDCl_3_) δ:** 173.15 (C-1), 163.52 (C-22´), 141.06 (C-21´), 135.83 (C-23´), 131.89 (C-26´), 123.67 (C-25´), 114.42 (C-24´), 65.03 (C-1´), 48.51 (CH_2_), 46.72 (CH_2_), 34.92 (CH_2_), 31.42 (CH_2_), 30.18 (CH_2_), 29.69 (29 × CH_2_), 29.34 (CH_2_), 27.07 (CH_2_), 22.68 (CH_2_), 14.10 (C-20).

**HR-MS *m/z*** 723.5420 **[M+K]^+^** (calcd. For C_46_H_84_O_3_K, 723.5420).

***Marsdenia tenacissima***

**Compound 1: Marsdenoside D** (Deng et al. 2005)

**^1^H NMR** (CDCl3): δ 4.83 (1H, d, J = 10.0 Hz, H12a), 4.80 (1H, d, J = 8.3 Hz, Allo-H-1), 4.60 (1H, dd, J = 9.6, 1.7 Hz, Ole-H-1), 3.88 (1H, t, J = 10.0 Hz, H11b), 3.80 (1H, t, J = 2.5 Hz, Allo-H-3), 3.67 (3H, s, Allo-3-OCH3), 3.64 (1H, m, H-3), 3.57(1H, m, Allo-H-5), 3.48 (1H, br d, J = 9.0 Hz, Allo-H-2), 3.40 (1H, m, Ole-H-3), 3.38 (3H, s, Ole3-OCH3), 3.37 (2H, m, Ole-H-4, 5), 3.18 (1H, dd, J = 9.6, 2.5 Hz, Allo-H-4), 2.92 (1H, br d, J = 7.3 Hz, H-17b), 2.33 (1H, ddd, J = 11.8, 4.5, 2.0 Hz, Ole-He-2), 2.21 (3H, s, 21-CH3), 1.71 (1H, d, J = 10.0 Hz, H-9), 1.50 (1H, m, Ole-Ha-2), 1.37 (3H, d, J = 4.9 Hz, Ole-6-CH3), 1.26 (3H, d, J = 6.2 Hz, Allo-6-CH3), 1.16 (3H, d, J = 7.0 Hz, 50 -CH3), 1.06 (3H, s, 19-CH3), 1.05 (3H, s, 18-CH3), 0.91 (3H, t, J = 7.4 Hz, 40 -CH3).

**^13^C-NMR:**

^13^C NMR (100 MHz, CDCl_3_) δ 210.7 (C-20), 77.2 (C-12), 76.8 (C-3), 71.9 (C-14), 67.6 (C-11), 66.9 (C-8), 60.0 (C-17), 52.6 (C-9), 45.8 (C-13), 44.2 (C-5), 39.1 (C-10), 38.4 (C-1), 34.8 (C-4), 31.9 (C-7), 30.1 (C-21), 29.1 (C-2), 26.9 (C-15), 26.6 (C-6), 25.0 (C-16), 16.7 (C-18), 12.6 (C-19).

Bu: 176.7 (C-1’), 41.3 (C-2’), 25.8 (C-3’), 15.1 (C-5’), 11.4 (C-4’).

Sugar unit: Ole: 97.1 (C-1), 79.2 (C-4), 78.8 (C-3), 71.4 (C-5), 55.6 (3-OH), 36.2 (C-2), 18.6 (C-6).

Allo: 99.1 (C-1), 81.0 (C-3), 72.9 (C-4), 71.6 (C-2), 71.3 (C-5), 61.9 (3-OH), 17.9 (C-6).

**HR-MS *m/z*** 775.4246 [M+Na]^+^, (calcd. [C_40_H_64_O_13_Na] 775.4245).

**Compound 2: Tenacissimoside B** (Ahmad and Basha, 2010)

**^1^HNMR:** (CDCl_3_, 400 MHz) : δ 7.93 (2H, dd, J=8.5, 1.3 Hz, H-3, H-7 of Benz), 7.51 (1H, t, J=7.3 Hz, H-5 of Benz), 7.39 (2H, t, J=7.5 Hz, H-4, H-6 of Benz), 5.57 (1H, t, J=10.1 Hz, H-11E), 5.09 (1H, d, J=10.1 Hz, H-12a), 4.76 (1H, d, J=8.4 Hz, H-1 of Dma), 4.53 (1H, dd, J=8.0, 1.4 Hz, H-1 of Ole), 3.63 (3H, s, OCH3 of Dma), 3.34 (3H, s, OCH3 of Ole), 2.93 (1H, d, J=7.3 Hz, H-17), 2.27 (1H, dd, J=10.1, 3.2 Hz, H-2 of Ole), 2.18 (3H, s, H-21), 1.57 (3H, s, OCOCH_3_), 1.31 (3H, J=5.3 Hz, H-6 of Dma), 1.22 (3H, d, J=6.0 Hz, H-6 of Ole), 1.12 (3H, s, H-18), 1.09 (3H, s, H-19).

**^13^C NMR:** (CDCl_3_, 100 MHz): δ 210.287 (C-20), 75.943 (C-3), 75.157 (C-12), 71.772 (C-14), 70.030 (C-11), 66.815 (C-8), 59.707 (C-17), 51.856 (C-9), 46.169 (C-13), 43.876 (C-5), 39.455 (C-10), 32.142 (C-15), 37.859 (C-1), 35.185 (C-4), 30.191 (C-21), 29.656 (C-2), 27.240 (C-6), 27.178 (C-16), 25.232 (C-7), 16.853 (C-18), 13.142 (C-19), Benz 170.620 (OCOCH3), 166.186 (C-1), 133.609 (C-5), 130.738 (C-2), 129.862 (C-3), 129.862 (C-7), 129.087 (C-4), 129.087 (C-6), 20.260 (OCOCH3).

**HR-MS *m/z*** 815.4218 [M+H]^+^ (calcd for C_44_H_63_O_14_, 815.4218).

***Nerium oleander***

**Compound 1: Ocotillol** (Tanaka et al. 1993)

**^1^HNMR:** (CDCl_3_, 400 MHz): δ 3.73 (IH, t, *J*=7.3 Hz, H-24), 3.20 (1H, dd, *J*=11.5 and 4.5 Hz, H-3a), 1.13 (3H, s, H-26), 1.21 (3H, s, H-27), 1.12 (3H, s, H-21), 0.97 (3H, s, H-28), 0.95 (3H, s, H-18), 0.87 (3H, s, H-30), 0.84 (3H, s, H-19), 0.77 (3H, s, H-29).

**^13^CNMR:** (CDCl_3_, 100 MHz): 86.44 (C-20), 83.31 (C-24), 78.95 (C-3), 71.43 (C-25), 55.85 (C-5), 50.80 (C-9), 50.08 (C-14), 49.54 (C-17), 42.97 (C-13), 40.38 (C-8), 39.05 (C-1), 38.96 (C-4), 37.18 (C-10), 35.67 (C-22), 35.29 (C-7), 31.48 (C-15), 28.03 (C-28), 27.43 (C-2, C-16, C-27), 26.13 (C-23), 25.74 (C-12), 24.28 (C-26), 23.55 (C-21), 21.59 (C-11), 18.30 (C-6), 16.47 (C-30), 16.25 (C-19), 15.47 (C-18), 15.34 (C-29).

**HR-MS *m/z*** 483.3814 [M+Na]^+^ (calcd for C_30_H_52_O_3_Na, 483.3814).

**Compound 2: Odoroside A** (Ahmad and Basha 2010, Abe et al. 1996)

**^1^HNMR:** (CDCl_3_, 400 MHz): δ 6.13 (IH, br s, H-22), 5.31 (1H, dd, *J*= 18, 2Hz, H-21a), 5.21 (1H, s, 14-OH), 5.03 (1H, dd, *J*= 18, 2Hz, H-21b), 4.75 (1H, dd, *J*= 10, 2Hz, H-1’), 4.33 (1H, br s, H-3), 3.90 (lH, brs, H-4'), 3.56, (1H, qd, *J*=6, 1Hz, H-5'), 3.42 (1H, ddd, *J* = 12, 4, 3 Hz, H-3'), 3.39 (3H, s, 3'-OMe), 2.80 (IH, dd, *J*=9, 4Hz, H-17), 1.55 (3H, d, *J*=6Hz, H-6'), 1.02 (3H, s, H-19), 0.91 (3H, s, H-18).

**^13^CNMR:** (CDCl_3_, 100 MHz): δ 174.5 (C-23), 174.3 (C-20), 117.4 (C-22), 73.4 (2 C-1), 72.5 (C-3), 85.5 (C-14), 50.8 (C-17), 49.5 (C-13), 41.8 (C-8), 40.0 (C-12), 36.3 (C-5), 35.7 (C-9), 35.1 (C-10), 33.0 (C-15), 30.1 (C-4), 29.8 (C-1), 26.9 (C-16), 26.6 (C-2), 26.6 (C-6), 23.6 (C-19), 21.4 (C-11), 21.1 (C-7), 15.7 (C-18), Din 97.6 (C-1), 77.9 (C-3), 70.3 (C-5), 67.1 (C-4), 55.6 (OCH_3_), 32.0 (C-2), 16.8 (C-6).

**HR-MS *m/z*** 519.3322 [M+H]^+^ (calcd for C_30_H_47_O_7_, 519.3322).

**Compound 3: β-Anhydroepidigitoxigenin** (Huq et al. 1999)

**^1^HNMR:** (400 MHz, DMSO-d_6_) δ 6.04 (1H, d, *J*=1.8 Hz, H-22), 5.18 (1H, d, *J*=1.8 Hz, H-15), 4.90 (dd, *J*= 17.4, 1.8 Hz, H-21a), 4.86 (dd, *J*= 17.4 Hz, 1.8, H-21b), 4.45 (br s, 1H, , H-3-OH), 3.38 (1H, m, H-3), 2.78 (1H, t, *J*=9 Hz, H-17), 2.49 (1H, m, H-16b), 2.35 (1H, d, *J*=9 Hz, H-16a), 1.96-1.90 (2H, m, H-12a, 18), 1.85 (1H, m, H-7a), 1.68-1.60 (3H, m, H-1b, 2a, 11a), 1.42-1.40 (2H, m, H-4b, 7b), 1.28-1.23 (5H, m, H-2b, 6a, 6b, 11b, 12b), 1.18 (1H, m, H-4a), 0.90 (1H, m, H-1a).

**^13^CNMR:** (100 MHz, DMSO-d_6_) 173.7 (C-23), 172.1 (C-20), 153.6 (C-14), 116.5 (C-15), 115.2 (C-22), 73.3 (C-21), 69.3 (C-3), 53.3 (C-9), 51.6 (C-17), 47.9 (C-13), 43.8 (C-5), 40.3 (C-12), 38.0 (C-4), 36.6 (C-1), 35.3 (C-10), 34.7 (C-8), 33.1 (C-16), 31.2 (C-2), 29.6 (C-7), 28.0 (C-6), 21.3 (C-11), 17.9 (C-18), 11.8 (C-19).

**HR-MS *m/z*** 356.2351 [M+H]^+^ (calcd for C_23_H_33_O_3_, 356.2351).

***Rauvolfia serpentina***

**Compound 1: 3-Hydroxysarpagine** (Rukachaisirikul et al. 2017)

**^1^H NMR** (400 MHz, CDCl_3_ + CD_3_OD (1:1): δ 7.82 (1H, brs, NH), 7.15 (1H, d, *J*=8.6 Hz, H-12), 6.81 (1H, brs, H-9), 6.68 (1H, d, *J*=8.6 Hz, H-11), 5.59 (1H, br d , *J*=6.4 Hz, H-19), 3.95 (1H, d, *J*=15.8 Hz, Hb-21), 3.86 (1H, d, *J*=15.8 Hz, Ha-21), 3.50 (2H, d, *J*=7.2 Hz, H-17), 3.28 (1H, H-5), 3.14 (1H, dd, *J*=16.1, 4.6 Hz, Hb-6), 3.00 (1H, brs, H-15), 2.83 (1H, d, *J*=16.1 Hz, Ha-6), 2.30 (1H, t, *J*=11.6 Hz, Hb-14), 2.03 (1H, d, *J*=7.2 Hz, H-16), 1.95 (1H, brd, *J*=14.8 Hz, Ha-14), 1.67 (3H, d, *J*=5.6 Hz, H-18).

**^13^C NMR** (100 MHz, CDCl_3_ + CD_3_OD (1:1)): δ 151.6 (C-10), 135.4 (C-2), 133.1 (C-13), 130.0 (C-20), 128.5 (C-8), 121.7 (C-19), 112.9 (C-12), 112.7 (C-11), 103.3 (C-9), 102.9 (C-7), 84.7 (C-3), 64.1 (C-17), 57.8 (C-5), 55.5 (C-21), 44.2 (C-16), 33.0 (C-14), 27.7 (C-15), 26.7 (C-6), 13.1 (C-18).

**HR-MS *m/z*** 327.1709 [M+H]^+^ (calcd for C_19_H_23_N_2_O_3_, 327.1709).

**Compound 2: Sarpagine** (Rukachaisirikul et al. 2017)

**^1^H NMR** (400 MHz, CDCl_3_ + CD_3_OD (1:1)): δ 7.82 (1H, brs, NH), 7.15 (1H, d, *J*=8.6 Hz, H-12), 6.81 (1H, brs, H-9), 6.68 (1H, d, *J*=8.6 Hz, H-11), 5.59 (1H, br d , *J*=6.4 Hz, H-19), 4.63 (1H, d, *J*=9.6 Hz, H-3), 3.95 (1H, d, *J*=15.8 Hz, Hb-21), 3.86 (1H, d, *J*=15.8 Hz, Ha-21), 3.50 (2H, d, *J*=7.2 Hz, H-17), 3.28 (1H, H-5), 3.14 (1H, dd, *J*=16.1, 4.6 Hz, H-6b), 3.00 (1H, br s, H-15), 2.83 (1H, d, *J*=16.1 Hz, H-6a), 2.30 (1H, t, *J*=11.6 Hz, Hb-14), 2.03 (1H, d, *J*=7.2 Hz, H-16), 1.95 (1H, br d, *J*=14.8 Hz, Ha-14), 1.67 (3H, d, *J*=5.6 Hz, H-18).

**^13^C NMR** (100 MHz, CDCl_3_ + CD_3_OD (1:1): δ 151.6 (C-10), 135.4 (C-2), 133.1 (C-13), 130.0 (C-20), 128.5 (C-8), 121.7 (C-19), 112.9 (C-12), 112.7 (C-11), 103.3 (C-9), 102.9 (C-7), 64.1 (C-17), 57.8 (C-5), 52.7 (C-3), 55.5 (C-21), 44.2 (C-16), 33.0 (C-14), 27.7 (C-15), 26.7 (C-6), 13.1 (C-18).

**HR-MS *m/z*** 311.1681 [M+H]^+^ (calcd for C_19_H_23_N_2_O_2_, 311.1681).

***Tabernaemontana divaricata***

**Compound 1: 5-Oxocoronaridine** (Liu et al. 2016)

**^1^H NMR** (CDCl_3_ , 400 MHz), δ 7.81 (1H, brs, N-H), 7.47 (1H, dd, *J*=1.2, 7.5 Hz, H-9), 7.24 (1H, dd, *J*=1.2, 6.3 Hz, H-12), 7.14 (1H, ddd, *J*=1.2, 6.3, 7.2 Hz, H-11), 7.08 (1H, ddd, *J*=1.2, 7.2, 7.5 Hz, H-10), 3.71 (3H, s, CO2 Me), 3.56 (1H, bs, H-21), 3.39 (1H, m, H-6b), 3.21 (1H, m, H-6a), 2.90 (1H, dd, *J*=3.9, 8.7 Hz, H-3b), 2.80 (1H, d, *J*=8.7 Hz, H-3a), 2.57 (1H, ddd, *J*=2.4, 5.7, 14.1 Hz, H-17b), 1.91 (1H, m, H-17a), 1.88 (1H, m, H-14), 1.74 (1H, m, H-15b), 1.55 (1H, m, H-19b), 1.45 (1H, m, H-19a), 1.33 (1H, m, H-20), 1.13 (1H, m, H-15a), 0.90 (3H, t, *J*=7.5 Hz, H-18).

**^13^C NMR** (CDCl_3_, 100 MHz), δ 175.9 (CO_2_ Me), 136.7 (C-2), 135.5 (C-13), 128.9 (C-8), 122.0 (C-11), 119.3 (C-10), 118.5 (C-9), 110.4 (C-7), 110.4 (C-12), 57.4 (C-21), 55.0 (C-16), 169.82 (C-5), 52.5 (CO2 Me), 51.5 (C-3), 39.1 (C-20), 36.4 (C-17), 31.9 (C-15), 27.3 (C-14), 26.6 (C-19), 22.0 (C-6), 11.5 (C-18).

**HR-MS *m/z*** 370.1893 [M+H_2_O]^+^ (calcd for C_21_H_26_N_2_O_4_, 370.1893).

**Compound 2: 19-Hydroxyconopharyngine** (Zocoler et al. 2005)

**^1^H NMR** (CDCl_3_, 400 MHz), δ 7.59 (1H, brs, N-H), 6.90 (1H, s, H-9), 6.78 (1H, s, H-12), 3.92 (3H, s, 11-OMe), 3.89 (3H, s, 10-OMe), 3.71 (3H, s, CO_2_ Me), 3.53 (1H, brs, H-21), 3.37 (1H, m, H-6b), 3.21 (1H, m, H-6a), 3.11 (1H, m, H-5b), 2.97 (1H, m, H-5a), 2.90 (1H, m, H-3b), 2.81 (1H, d, *J*=8.4 Hz, H-3a), 2.54 (1H, ddd, *J*= 2.4, 5.7, 14.1 Hz, H-17b), 1.90 (1H, m, H-17a), 1.87 (1H, m, H-14), 1.73 (1H, m, H-15b), 2.54 (1H, m, H-19), 1.31 (1H, m, H-20), 1.12 (1H, m, H-15a), 0.89 (3H, t, *J*=7.5 Hz, H-18).

**^13^C NMR** (CDCl_3_, 100 MHz), δ 175.9 (CO_2_ Me), 154.2 (C-11), 152.3 (C-10), 136.7 (C-2), 135.5 (C-13), 128.9 (C-8), 118.5 (C-9), 110.4 (C-7), 110.4 (C-12), 62.8 (C-19), 57.4 (C-21), 56.2 (OMe-C11), 55.6 (OMe-C10), 55.0 (C-16), 53.1 (C-5), 52.5 (CO_2_ Me), 51.5 (C-3), 39.1 (C-20), 36.4 (C-17), 31.9 (C-15), 27.3 (C-14), 22.0 (C-6), 11.5 (C-18).

**HR-MS *m/z*** 453.1792 [M+K]^+^ (calcd for C_23_H_30_KN_2_O_5_, 453.1792).
